# Atlas-Guided Nanocarrier Strategies Targeting Spatial NTRK2/MAPK Signaling in EGFR-TKI-Resistant Niches of Esophageal Squamous Cell Carcinoma

**DOI:** 10.3390/pharmaceutics18020181

**Published:** 2026-01-30

**Authors:** Xiusen Zhang, Xudong Zhang, Xing Jin, Shilei Zhang, Xin Zhao, Hairui Wang, Hui Wang, Lijun Deng, Wenchao Tang, Qizhi Fu, Shegan Gao

**Affiliations:** 1 Key Laboratory of Microbiome and Esophageal Cancer Prevention and Treatment, The First Affiliated Hospital, and College of Clinical Medicine of Henan University of Science and Technology, Luoyang 471003, China; zhangxs@stu.haust.edu.cn (X.Z.); 240410200114@haust.edu.cn (X.Z.); 240410200103@stu.haust.edu.cn (S.Z.); 250410200169@stu.haust.edu.cn (H.W.); 2 National Key Laboratory of Genetic Evolution & Animal Models, Kunming Institute of Zoology, Chinese Academy of Sciences, Kunming 650201, China; 3 Zhengzhou Center for Disease Control and Prevention, Zhengzhou Health Inspection Institute, Zhengzhou 450003, China; 4 Department of Critical Care Medicine, The First Affiliated Hospital, and College of Clinical Medicine of Henan University of Science and Technology, Luoyang 471003, China; 5 Institute of Organoid on Chip and Drug Translation Research, Henan Academy of Sciences, Zhengzhou 450046, China

**Keywords:** esophageal squamous cell carcinoma, epidermal growth factor receptor-tyrosine kinase inhibitor resistance, spatial omics, NTRK2/MAPK signaling axis, intelligent nanocarrier systems, atlas-driven precision therapy

## Abstract

Esophageal squamous cell carcinoma (ESCC) represents a major therapeutic challenge due to the rapid development of resistance to epidermal growth factor receptor-tyrosine kinase inhibitors (EGFR-TKIs). Recent evidence highlights that this resistance is driven not only by genetic mutations but also by spatial heterogeneity of tumor microenvironments and compensatory signaling mechanisms. In this review, we propose a “spatial-signaling-intervention” framework with a particular focus on the NTRK2/MAPK signaling axis, which plays dual roles in signaling compensation and immune evasion. By integrating spatial multi-omics, proteomics, and AI-assisted topological modeling, three resistant niches are identified: (1) cancer stemness-enriched zones, (2) MAPK hyperactive islands, and (3) immune-cold regions. Based on this atlas, we design precision nanotherapeutic platforms, including responsive, dual-target, and feedback-loop nanocarriers, to selectively modulate resistant spatial niches. Preclinical validation in patient-derived xenografts and organoid models further demonstrates the translational potential of these strategies. This work provides a conceptual and technological roadmap for overcoming EGFR-TKI resistance in ESCC. Atlas-guided nanocarrier systems offer a promising avenue for spatially targeted and feedback-responsive therapy, highlighting the role of pharmaceutics in advancing precision oncology.

## 1. Introduction

Esophageal squamous cell carcinoma (ESCC) is the most prevalent histological subtype of esophageal cancer globally, with persistently high incidence and mortality rates, particularly in East Asian countries such as China, Japan, and South Korea [1,2,3]. According to estimates from GLOBOCAN 2022, there were approximately 511,054 newly diagnosed esophageal cancer cases and 445,391 related deaths worldwide in 2022. Of these, China accounted for 224,012 new cases (approximately 43.8%) and 187,467 deaths (approximately 42.1%), with an age-standardized incidence rate (ASIR) of 8.30 per 100,000 and an age-standardized mortality rate (ASMR) of 6.70 per 100,000 [4]. Despite recent advancements in molecular subtyping, immunotherapy, and targeted therapies, which have gradually shaped a more structured diagnostic and therapeutic framework for ESCC, overall patient prognosis remains poor, with the median survival time still limited [5,6].

**Figure 1:**
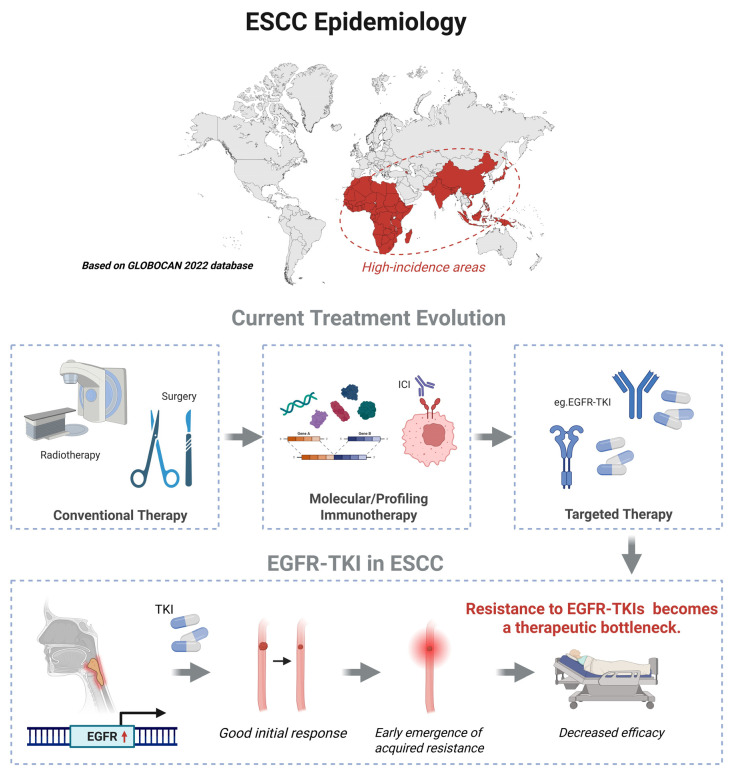
Overview of the Current Application and Challenges of EGFR-TKI Therapy in ESCC. Created with BioRender.com. T, J. (2025) https://BioRender.com/n3sy5wj. Note: High-incidence regions of ESCC are primarily located in Eastern Africa, East Asia, Southern Africa, and South-Central Asia. The current treatment paradigm is shifting from conventional approaches to molecular subtype-guided immunotherapy and targeted therapies. Although patients with high EGFR expression initially respond well to EGFR-TKI treatment, most develop acquired resistance within several months, resulting in reduced therapeutic efficacy and limited survival benefit. EGFR-TKI resistance has thus become a major therapeutic bottleneck.

Epidermal growth factor receptor-tyrosine kinase inhibitors (EGFR-TKIs), a class of targeted therapeutic agents, have demonstrated initial treatment responses in a subset of ESCC patients with high EGFR expression [7,8,9]. However, in clinical practice, the majority of patients developed acquired resistance within several months of EGFR-TKI administration, which significantly compromised long-term efficacy and remains one of the primary challenges in the development of targeted therapies for esophageal cancer (Figure 1) [10,11].

The mechanisms underlying EGFR-TKI resistance are highly complex, encompassing not only secondary mutations within the EGFR gene itself and ligand-dependent enhancement, but also the activation of various bypass signaling pathways, such as MET, HER2, and FGFR. Additionally, processes including epithelial–mesenchymal transition (EMT), alterations in apoptotic regulation, and the selective enrichment of cancer stem cell (CSC) populations further contribute to resistance [12,13]. However, explaining these mechanisms solely through the lens of linear pathway activation or genetic mutation has proven insufficient to account for the heterogeneous resistance phenotypes observed in clinical settings [14,15].

Recent multi-omics studies have highlighted that the development of resistance is not merely the result of intracellular signaling alterations, but is also driven by spatial heterogeneity within the tumor microenvironment (TME) [16,17]. For instance, combined single-cell RNA sequencing (scRNA-seq) and spatial transcriptomic (ST) analyses have revealed that EGFR activity is significantly higher at the tumor periphery than at the core, accompanied by the upregulation of immune evasion-related pathways [18]. Experimental evidence has further indicated that, under the selective pressure of EGFR-TKI treatment, tumor cells located at the margins exhibit enhanced survival capacity and rapidly evolve into resistant subclones. These findings suggest a spatially correlated relationship between asymmetric signal activation and the clonal selection of resistant populations [19,20,21].

In addition to the structural heterogeneity arising from spatial distribution, the dynamic topological architecture of downstream EGFR signaling networks represents another central mechanism driving therapeutic resistance [22,23]. Traditional models depicting the EGFR pathway as a linear cascade through either the RAS/MAPK or PI3K/AKT axis fail to account for the observed “inhibition-rebound” fluctuations in signaling activity [24]. Recent studies in network biology and systems pharmacology have demonstrated that the EGFR signaling cascade constitutes a complex feedback-branch-redundancy-coupled network, comprising parallel and compensatory modules such as PI3K, STAT3, JAK/IL6, and TrkB, along with feedback amplification circuits [24,25,26]. Upon EGFR inhibition, these “bypass routes” can be rapidly activated to sustain cellular viability [27,28,29]. This phenomenon of signaling rewiring has been validated in multiple primary ESCC cell lines through phosphoproteomic profiling and dynamic metabolic tracing, underscoring its pivotal role in the development of acquired resistance [30,31].

Given the interplay between spatial heterogeneity and signaling topological complexity, monotherapeutic approaches targeting a single molecular entity are insufficient to overcome the multifaceted resistance landscape [32,33]. Consequently, developing an integrative strategy capable of simultaneously identifying critical spatial zones, deciphering functional signaling axes, and precisely delivering therapeutic agents has emerged as a key direction in the field [34]. On one front, spatial omics platforms are employed to delineate therapeutic “blind spots” and signaling “hot islands” within tumor tissues; on the other, network control theory and dynamic modeling are applied to extract “vulnerable control nodes” from intricate signaling networks, thereby optimizing target combination strategies [35,36]. Building upon these insights, responsive nanocarrier systems are developed in conjunction with spatial prediction models, enabling site-specific delivery to high-risk niches and establishing closed-loop feedback control mechanisms [37,38,39].

It is important to note that the term “spatial heterogeneity” in this study refers not only to the spatial clustering of specific cell types but, more critically, to the asymmetric distribution of signaling functional states within the native structural context of the tissue. The term “spatial niches” denotes functionally distinct microregions within the tumor that possess defined spatial coordinates, signaling axis configurations, and microenvironmental characteristics. Examples include stemness-enriched zones, isolated MAPK signaling islands, and immune-cold regions—areas that frequently correspond to “low-response zones” or “therapeutic blind spots” under EGFR-TKI treatment [36].

Unlike existing models that primarily interpret EGFR-TKI resistance from the perspective of gene mutations or single signaling pathways, recent studies integrating spatial omics and signaling network analysis have proposed a novel “Spatial–Signaling–Intervention (SSI)” framework, which incorporates spatial topology, NTRK2/MAPK signaling coupling, and closed-loop nano-intervention systems into a unified analytical paradigm. The innovation of this framework lies in three key aspects. First, it expands the understanding from “mutation-driven mechanisms” to “spatial topological constraints.” Using spatial transcriptomics and multi-omics analyses, previous studies have clearly revealed the spatial distribution of functional compartments within tumor tissues, such as CSC-enriched regions, MAPK hyperactive islands, and immune-cold zones, and their associations with drug resistance [40,41]. Second, it unifies signaling compensation and immune evasion into a spatially defined NTRK2/MAPK hub. Experimental and spatial evidence in ESCC and related solid tumors have indicated that the NTRK2-MAPK/ERK axis contributes to the development of EGFR-TKI resistance [11]. Third, by employing AI-based topological modeling, the framework enables a conceptual transformation from a “drug–target” to a “drug–map” paradigm. Recent reviews and methodological studies have emphasized the use of multimodal spatial omics data as interpretable inputs for predictive modeling and therapeutic navigation [42]. Compared with conventional models that are based on mutational spectra, linear pathways, or macroscopic microenvironmental descriptions [41,43], the SSI framework achieves three major theoretical and methodological advancements. It introduces the spatial topological dimension by applying mature spatial algorithms such as Hotspot and SpaGCN to identify functional compartments and ecological niche boundaries, thereby transforming the analytical focus from “homogeneous pathways” to “spatial networks” [44,45]. It provides spatially localized validation of the NTRK2/MAPK axis, demonstrating its co-enrichment in CSC-dense and immune-cold regions as well as its compensatory reactivation following EGFR inhibition [11]. Finally, it establishes a spatially guided intervention strategy in which responsive and monitoring-integrated nanosystems enable a closed-loop process of “map identification → target selection → delivery feedback.” The feasibility of these stimuli-responsive and in vivo monitoring strategies has been demonstrated in recent studies [46,47]. Collectively, the SSI framework elevates EGFR-TKI resistance research from a “one-dimensional mutation–pathway” model to a multidimensional “spatial–signaling–functional” system. By integrating multimodal spatial omics with AI-driven modeling, it provides a systematic paradigm for precision spatial intervention (Supplementary Table S1) [48].

**Figure 2:**
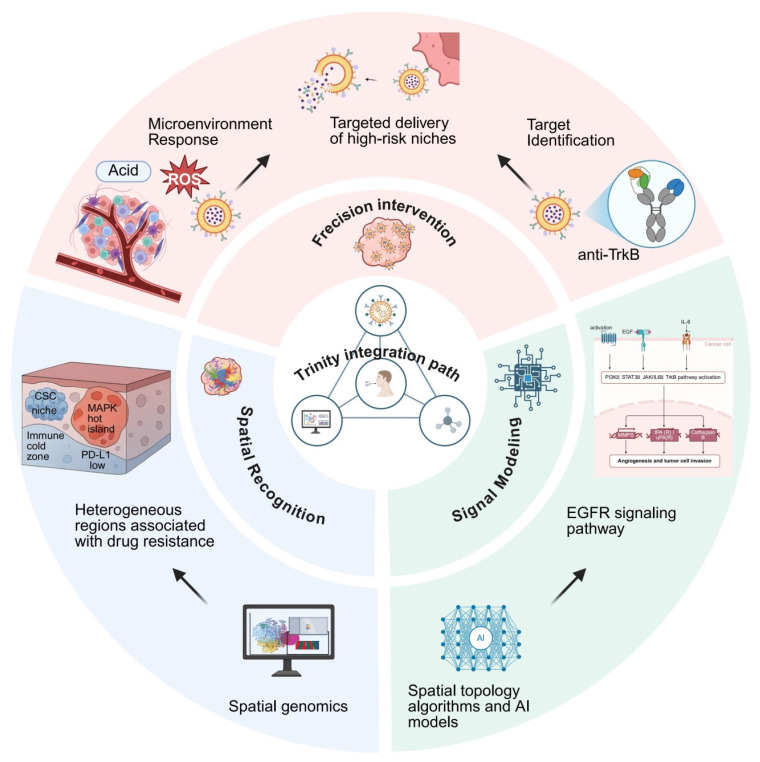
Integrated Framework of “Spatial Identification-Signal Modeling-Precision Intervention”. Created with BioRender.com. T, J. (2025) https://BioRender.com/xride4s. Note: Compared with conventional models centered on genetic mutations or single-pathway regulation, this framework introduces a three-step process consisting of spatial topology recognition → NTRK2/MAPK hub localization → closed-loop nanointervention. By applying spatial omics to identify heterogeneous tumor regions (such as CSC-enriched zones, MAPK “hot islands,” and immunologically cold regions), the framework integrates signal pathway modeling with AI-driven node prioritization to pinpoint key regulatory hubs. This facilitates target identification, precision delivery of responsive nanomedicines, and microenvironmental modulation, establishing a spatially targeted intervention strategy for EGFR-TKI resistance in esophageal cancer.

Taken together, the essence of EGFR-TKI resistance in ESCC lies in a complex adaptive mechanism driven by spatially regulated subclonal selection and signaling network rewiring [41,42]. This review adopts an integrative framework based on the triad of “spatial identification—signaling modeling—precision intervention” to systematically examine the current understanding of resistance mechanisms, key signaling axes, and therapeutic strategies. Furthermore, we propose a novel spatially targeted therapeutic model centered on the NTRK2/MAPK axis as a core connecting node. This model establishes a theoretical foundation spanning from atlas-based spatial recognition to intelligent nanocarrier delivery, providing a reference framework for implementing precision spatial interventions in esophageal cancer [49,50] (Figure 2).

## 2. Mechanisms of Egfr-Tki Resistance Involving Molecular Mutations and Spatial Heterogeneity

### 2.1. The Role of Egfr and Its Signaling Pathways in Escc

EGFR is a type I transmembrane tyrosine kinase receptor that is widely expressed in epithelial tissues and plays a central role in regulating cell proliferation, differentiation, migration, and apoptosis [11,51]. Multi-omics analyses have revealed that EGFR is markedly overexpressed and frequently amplified in ESCC, whereas classical activating mutations—such as L858R and exon 19 deletions—are relatively rare. This pattern contrasts sharply with that observed in non-small cell lung cancer (NSCLC) [11,51].

**Figure 3:**
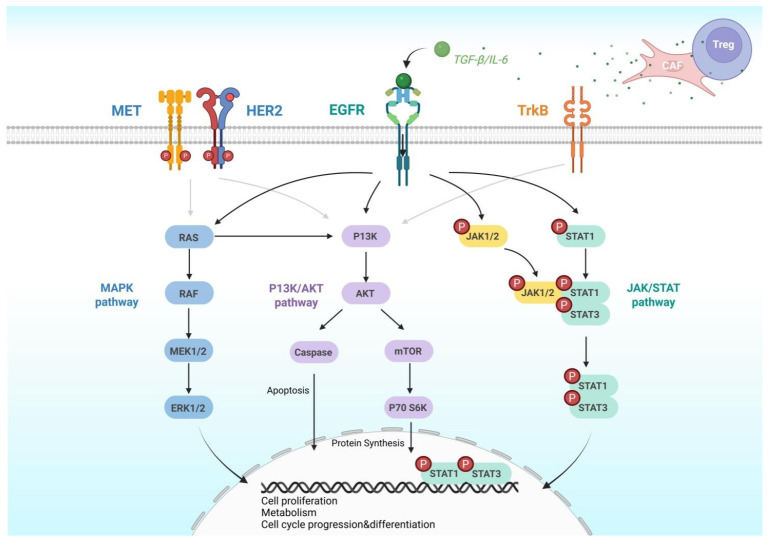
Schematic Diagram of the Three Major Downstream Signaling Pathways of EGFR. Created with BioRender.com. T, J. (2025) https://BioRender.com/izlqd3d. Note: (1) RAS-RAF-MEK-ERK (MAPK pathway); (2) PI3K-AKT-mTOR pathway; (3) JAK-STAT pathway.

Upon activation, EGFR transmits signals primarily through three major downstream pathways: (1) The RAS-RAF-MEK-ERK (MAPK) axis, which governs cell proliferation and invasion; (2) The PI3K-AKT-mTOR axis, responsible for anti-apoptotic signaling and metabolic regulation; (3) The JAK-STAT axis, which is involved in inflammation, immune modulation, and the maintenance of cellular stemness [52,53,54] (Figure 3).

In ESCC, regions of high EGFR expression are often associated with enhanced phosphorylation of these pathways—such as elevated levels of p-ERK and p-AKT—indicating a tendency toward co-activation among multiple signaling axes [51]. Consistently, animal models have demonstrated that EGFR-overexpressing ESCC cell lines exhibit accelerated tumor growth and increased metastatic potential, further supporting the oncogenic role of EGFR in this malignancy [11,51].

In addition to intracellular signaling, EGFR also engages in complex interactions with the TME [55,56]. In tissue regions exhibiting high EGFR activity, an increased density of cancer-associated fibroblasts (CAFs) was commonly observed, accompanied by upregulated expression of immunosuppressive factors such as TGF-β and IL-6 [57,58]. These factors not only promoted immune evasion but also reinforced EGFR expression and activation through the establishment of positive feedback loops [3,56]. Co-culture experiments further validated that EGFR-overexpressing ESCC cells, when cultured with CAFs or immunosuppressive cells, exhibited enhanced activation of the MAPK and PI3K pathways and demonstrated greater resistance to treatment [55,56,59].

In summary, EGFR in ESCC functions not only as a signaling initiator but also as a multidimensional regulatory hub that interlinks the TME, immune modulation, and signaling plasticity.

### 2.2. Current Status and Limitations of Egfr-Tki Application in Escc

**Figure 4:**
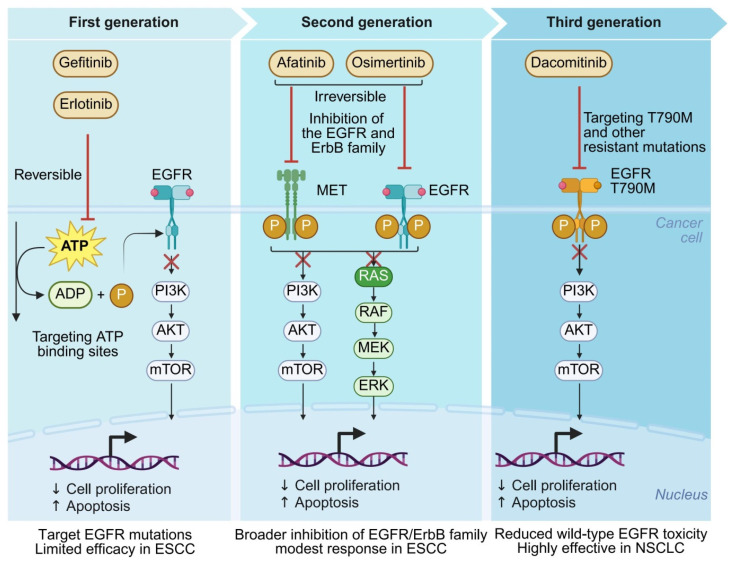
Developmental Timeline of EGFR-TKI Drugs and Response Variability in ESCC. Created with BioRender.com. T, J. (2025) https://BioRender.com/19q1ltj. Note: First-generation EGFR-TKIs are reversible inhibitors that block downstream PI3K/AKT/mTOR signaling by competitively binding to the ATP site. Second-generation inhibitors are irreversible and target multiple members of the EGFR and ErbB families, with partial suppression of the RAS/RAF/MEK/ERK cascade. Third-generation inhibitors are specifically optimized to overcome acquired resistance mutations, such as T790M, and have demonstrated significantly improved efficacy in EGFR-mutant populations.

EGFR-TKIs have undergone three generations of development: first-generation agents (Gefitinib, Erlotinib) act as reversible inhibitors targeting the ATP-binding site; second-generation inhibitors (Afatinib, Dacomitinib) are irreversible and simultaneously suppress multiple members of the ErbB family; third-generation inhibitors (Osimertinib) were specifically designed to overcome resistance mutations such as T790M [60] (Figure 4).

While these agents have been widely adopted in NSCLC, their application in ESCC has remained limited. Due to the rarity of EGFR-activating mutations in ESCC, targeted therapy strategies have primarily focused on patients with high EGFR expression rather than those harboring mutation-driven activation [3]. Several clinical studies have reported that Afatinib induces a partial response rate (objective response rate [ORR] ~15–20%) in ESCC patients with EGFR IHC 3+ expression and extends progression-free survival (PFS) in select cases [11]. In addition, the combination of Afatinib and Crizotinib (a MET inhibitor) demonstrated synergistic antitumor effects in patient-derived xenograft (PDX) models, supporting the feasibility of co-targeting bypass co-expression pathways [11,61].

However, the overall efficacy of EGFR-TKI monotherapy in ESCC remains limited. Key challenges include the lack of mutation-based screening criteria, high inter-patient heterogeneity, insufficient capacity of single-agent therapy to counteract multi-pathway resistance, immunosuppressive barriers constructed by the TME that hinder drug efficacy, and the absence of resistance mechanisms aligned with current patient stratification systems [3]. As a result, therapeutic strategies are shifting from single-target approaches toward integrated models that emphasize “pathway coordination + subpopulation stratification + combinatorial intervention” [62]. Stratification frameworks based on spatial heterogeneity and signaling network topology are expected to provide a theoretical foundation and actionable targets for enhancing EGFR-TKI efficacy [41,42].

### 2.3. Genetic Mutations, Immune Remodeling, Cancer Stemness, and Spatial Context in Egfr-Tki Resistance Mechanisms

EGFR-TKI resistance mechanisms are highly heterogeneous and can be broadly categorized into three core pathways:

(1) On-target mutations and compensatory bypass activation: In ESCC, secondary mutations in EGFR—such as T790M and C797S—are relatively uncommon. Instead, resistance more frequently arises from compensatory activation of bypass pathways, including MET amplification, HER2 upregulation, and activation of AXL and IGF1R [32,63,64]. These signaling axes—such as RAS/MAPK, PI3K/AKT, and STAT3—can substitute for EGFR activity, thereby sustaining cell survival [65,66]. Preclinical studies have shown that combining Afatinib with Lapatinib (a HER2 inhibitor) or Crizotinib (a MET inhibitor) significantly enhances antitumor efficacy, supporting multi-target combination therapy as a promising approach to overcoming bypass-driven resistance [61,64].

(2) Cancer stemness-mediated resistance: Selective pressure from EGFR-TKIs can induce phenotypic shifts in tumor cells, leading to enhanced EMT and enrichment of cancer stem-like properties [67]. These transformed cells exhibit greater migratory capacity, drug resistance, and metastatic potential, and have been shown to display pronounced resistance in both PDX and organoid models [3,68]. Emerging evidence suggests that stemness-related signaling pathways, such as TrkB/NTRK2 and SOX2, are activated during EGFR-TKI treatment and participate in MAPK pathway compensation [11]. Their spatial distribution patterns will be elaborated in Section 4. Furthermore, the downregulation of the epithelial marker E-cadherin and upregulation of mesenchymal markers such as Vimentin are widely observed in resistant regions. Inhibiting upstream EMT regulators such as Twist or Slug partially restores sensitivity to EGFR-TKIs [69,70,71].

(3) Immune microenvironment remodeling and immune evasion: Following EGFR-TKI treatment, immunosuppressive factors such as TGF-β and IL-10 were upregulated within tumor tissues, promoting the enrichment of regulatory T cells (Tregs) and M2-polarized macrophages, thereby enhancing the tumor’s capacity for immune evasion [72,73]. In addition, activation of immune checkpoints such as PD-L1 and IDO1 significantly suppressed the cytotoxic function of CD8^+^ T cells, further impairing antitumor immune responses [43,74]. In organoid-immune cell co-culture systems, combination therapies involving EGFR-TKIs and inhibitors targeting CSF1R or IDO1 partially restored immune effector functions, highlighting the critical role of immune regulation in EGFR-TKI resistance [75,76,77]. The spatial distribution patterns of these associated pathways will be further analyzed in Section 4 to elucidate the relationship between immune suppression and spatial selectivity.

Although the phenotypic manifestations of the above three mechanisms differ markedly, they can all be attributed to canonical processes of signaling pathway rewiring and spatial niche reconstruction. The table below provides a systematic summary of the most extensively reported EGFR-TKI resistance mechanisms, representative signaling axes, and potential combinatorial intervention strategies (Table 1).

EGFR-TKI resistance is not the result of a single mutational event; rather, it constitutes a systemic process driven by the interplay of signaling axis rewiring, cellular state evolution, and selective pressures imposed by the spatial microenvironment [84,85,86].

**Figure 5:**
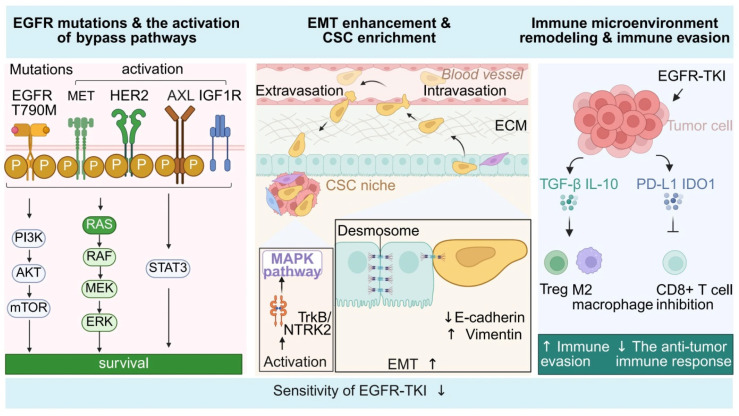
Schematic Diagram of the Ternary Mechanisms Underlying EGFR-TKI Resistance. Created with BioRender.com. T, J. (2025) https://BioRender.com/x736zja. Note: EGFR-TKI resistance can be broadly classified into three core mechanisms: (1) target mutations and activation of bypass signaling pathways; (2) enhanced EMT and enrichment of cancer stemness; and (3) remodeling of the immune microenvironment and immune evasion. These mechanisms collectively contribute to the decline in EGFR-TKI sensitivity and define a resistance ecosystem characterized by signal pathway reprogramming and spatial ecological remodeling.

Mechanism-specific explanations alone are no longer adequate in the context of spatial heterogeneity. Therefore, it is essential to integrate multi-omics data, spatial omics platforms, and functional modeling approaches to identify critical resistant regions and actionable targets, thereby laying the groundwork for subsequent spatially targeted intervention strategies (Figure 5).

## 3. Spatial Omics Foundations and Topological Modeling of Heterogeneity

### 3.1. Overview of Spatial Omics Technology Platforms (Visium, Cosmx, Stereo-Seq, etc.)

Spatial omics, serving as a bridge between tissue architecture and molecular information, has recently revolutionized research on tumor heterogeneity [55,59,87]. Unlike conventional bulk RNA sequencing (bulk RNA-seq) or scRNA-seq, spatial omics retains the spatial coordinates of cells, enabling the analysis of gene expression states within the context of the tissue microenvironment [59,88,89]. The Visium ST platform, developed by 10x Genomics, uses a predefined probe array to capture RNA and register tissue sections to obtain spatial information. It is currently one of the most widely adopted commercial platforms [90,91]. The CosMx Spatial Molecular Imager achieves subcellular resolution through multiple rounds of probe-based imaging, offering a balanced trade-off between spatial precision and target throughput [92,93,94]. Independently developed in China, the Stereo-seq platform utilizes nanobarcode labeling and DNA nanoball arrays to achieve spatial resolution beyond the subcellular level for the first time. This technology is particularly well-suited for studying complex, layered, and spatially heterogeneous solid tumors such as ESCC [95,96].

Each platform offers distinct advantages and limitations in terms of spatial resolution, tissue coverage, detection throughput, and operational convenience. Visium is well-suited for large tissue sections, with a spatial resolution of approximately 55 μm, making it ideal for visualizing global signaling pathway distribution patterns. It has already been applied to resistance atlas construction in tumors such as lung and liver cancers [97]. CosMx provides higher flexibility in target selection and image quality, making it appropriate for multimodal labeling and spatial proteogenomic co-detection studies [98,99]. Stereo-seq offers unique advantages in high-resolution and three-dimensional imaging and is particularly well-adapted for tracking longitudinal signaling changes from the basal to the luminal layers in esophageal tissue [87,100]. In ESCC, preliminary studies utilizing Stereo-seq identified MAPK signaling “islands,” hypoxic immune-exclusion zones, and TrkB-overexpressing hotspots, revealing a significant association between spatial niches and therapeutic response [101,102]. Integrating multiple platforms enables the generation of a comprehensive spatial expression atlas that captures both macroscopic tissue architecture and microscale functional hotspots, providing a multidimensional foundation for subsequent modeling and targeted intervention [55,59,87].

### 3.2. Topological Modeling Algorithms and Spatial Identification Tools

The acquisition of ST data represented only the first step; the core challenge lay in effectively modeling these high-dimensional datasets to interpret the biological significance underlying spatial heterogeneity, which is essential for achieving precision targeting [103]. Topological spatial algorithms aimed to identify statistically significant gene co-expression regions—termed functional spatial domains—and structurally characterize them through graph representations, spatial autocorrelation patterns, and adjacency networks [104,105]. Unlike traditional clustering approaches, these algorithms incorporated not only expression intensity but also spatial continuity, neighborhood proximity, and boundary structure detection [106,107,108]. The goals of topological modeling extended beyond classifi+cation to include prediction, identification of anomalous regions, and decoding of signal enrichment pathways. These capabilities made them particularly suitable for constructing complex, multi-regional resistance atlases (Table 2) [105,109,110].

Hotspot is an algorithm based on spatial autocorrelation, which computes local expression deviations for each gene within its neighborhood using a Gaussian kernel function to identify spatial co-expression modules [41,44,116]. In ESCC research, Hotspot has been applied to pinpoint TrkB/MAPK co-activation domains and to associate them with cancer stemness-related signaling expression [5,68].

Giotto is an R-based integrated platform that combines cell type annotation, adjacency relationship analysis, differential expression, and spatial visualization tools, making it well-suited for constructing structure-informed expression networks. In a spatial stratification study of ESCC organoids, Giotto successfully identified spatial coupling between signaling hubs and immune evasion centers [87,117].

**Figure 6:**
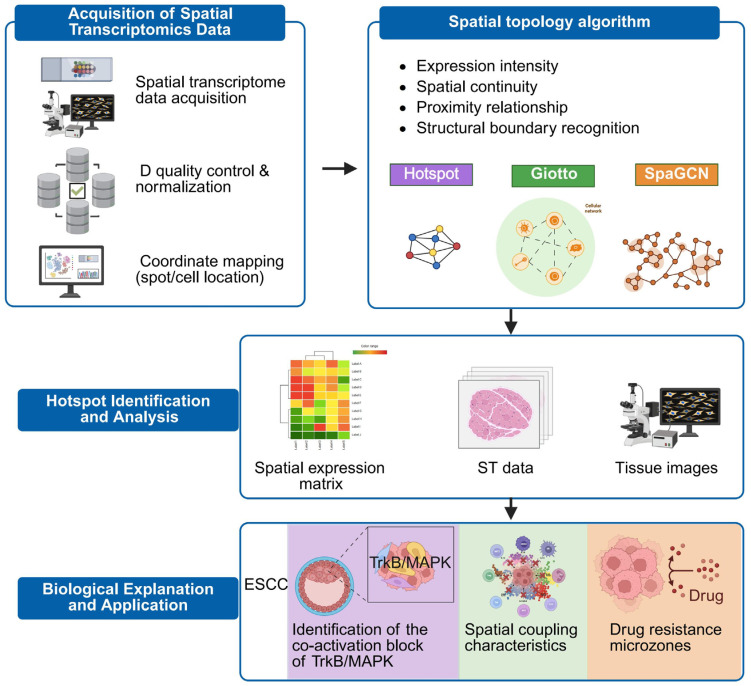
Workflow of Spatial Topological Modeling and Hotspot Region Identification. Created with BioRender.com. T, J. (2025) https://BioRender.com/q71v393. Note: Application of ST in ESCC involves the following steps: initial acquisition, quality control, and normalization of ST data; subsequent localization of cells/spots based on spatial coordinates; and topological modeling using algorithms that incorporate expression intensity, spatial continuity, neighborhood relationships, and structural boundaries. This process generates spatial expression matrices, tissue sections, and imaging data to identify significant functional hotspot regions. Finally, integration with ESCC cases enables biological interpretation and application, providing a rationale for drug delivery and precision intervention. ST: Spatial Transcriptomic; ESCC: Esophageal Squamous Cell Carcinoma.

SpaGCN is the first tool to incorporate graph convolutional networks (GCNs) into spatial omics analysis. It learns joint embedding vectors from gene expression profiles and spatial coordinates to uncover nonlinear regional expression patterns [45,118,119]. In highly heterogeneous tissues such as ESCC, SpaGCN proved capable of identifying spatial microdomains shaped by the interplay of multiple signaling sources, providing an algorithmic foundation for artificial intelligence (AI)-driven signaling prediction models (Figure 6) [120].

This study (i.e., the literature summarized in this review) involves data integration workflows across multiple spatial omics platforms. Recent spatial, single-cell, and spatial-transcriptomic studies have generally adopted standardized quality control and integration strategies [121]. For example, raw transcriptomic data are typically processed using official pipelines such as Space Ranger combined with custom scripts for alignment and counting. Quality control thresholds are usually set as follows: the number of detected genes per spot or cell ≥ 300, mitochondrial gene proportion ≤ 15%, and exclusion of blank or misclassified tissue-boundary regions [41]. At the transcriptional level, normalization is commonly performed using SCTransform (log1p transformation, variable feature number ≈ 3000), followed by vector scaling or cosine normalization within each section. To minimize batch effects across samples, cross-validation strategies using Harmony (theta ≈ 2, lambda ≈ 1) and limma are widely employed [122,123,124]. Spatial graph construction generally applies a parallel adjacency strategy combining k-nearest neighbors (kNN, k ≈ 8) and radius-based neighborhood adjacency (r ≈ 100 μm) to establish dual-domain networks integrating gene expression and spatial proximity. At the proteomic and metabolomic levels, ComBat or ComBat-seq is used for batch correction, whereas cross-modality alignment is achieved through the joint use of canonical correlation analysis (CCA) and mutual nearest neighbor (MNN) anchoring (anchor number ≈ 5000) [124,125,126]. Within a unified coordinate space, spatial co-expression modules are typically identified using Hotspot (Gaussian kernel σ ≈ 1.5 neighboring spots, FDR < 0.05) and Giotto (neighborhood radius ≈ 120 μm). In addition, SpaGCN (three-layer graph convolutional network, hidden dimension ≈ 64, epochs ≈ 500, learning rate ≈ 1 × 10^−3^, weight decay ≈ 1 × 10^−4^) is employed to learn integrated expression–spatial embeddings for refining the boundaries of resistant ecological niches and assessing regional coherence [44,45,112]. Further details are summarized in Supplementary Table S2.

### 3.3. Multimodal Integration and AI-Assisted Modeling

**Figure 7:**
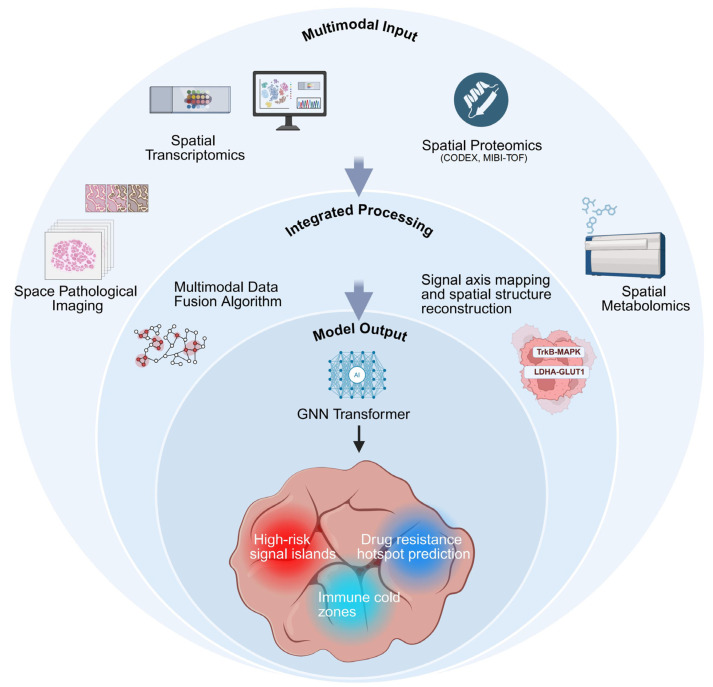
Integrated Framework of Multimodal Spatial Omics and AI-Based Modeling. Created with BioRender.com. T, J. (2025) https://BioRender.com/ca1kfsw. Note: Multimodal spatial omics data are integrated to elucidate tumor heterogeneity and predict resistance mechanisms. The outer layer consists of various input modalities, including ST, spatial proteomics, spatial metabolomics, and spatial pathological imaging. The middle layer applies multimodal data fusion algorithms to achieve feature alignment and atlas embedding, combined with signal axis mapping and spatial structure reconstruction. Representative examples include the TrkB-MAPK signaling axis and the LDHA-GLUT1 metabolic axis. The innermost layer employs AI models to generate functional spatial atlases, enabling the identification and annotation of high-risk signaling islands, immune-cold regions, and resistance hotspots, thereby providing a foundation for precise therapeutic interventions and optimization of treatment strategies.

As spatial technologies evolved toward multimodality, ST was no longer the sole analytical dimension [41]. Spatial proteomics platforms, such as CODEX and MIBI-TOF, provided spatial information on protein expression and activation states, while metabolomics technologies, including MALDI-IMS, revealed spatial patterns of tumor metabolic reprogramming [127,128]. In ESCC, MIBI-TOF-based labeling of proteins such as p-MEK and p-ERK enabled direct validation of MAPK pathway activity within TrkB activation zones. Meanwhile, the spatial enrichment of metabolic proteins such as lactate dehydrogenase A (LDHA) and glucose transporter 1 (GLUT1) was closely associated with immune exclusion and resistance to EGFR-TKIs [129,130,131]. The emerging research paradigm has shifted toward using ST as the central scaffold while integrating proteomic, metabolomic, and even histopathological imaging data to enable atlas-level modeling of signaling axes (Figure 7) [55,100,132].

The application of AI in spatial omics has progressed from auxiliary analysis to predictive modeling [133,134]. Deep learning architectures, particularly those based on graph neural networks (GNNs), have enabled the simultaneous integration of spatial coordinates, gene expression profiles, and tissue structural maps, thus facilitating multi-level data fusion [133,135,136]. Recent studies have developed spatial variational autoencoder models capable of automatically identifying immune-cold regions and classifying signaling hotspots [137]. In the context of EGFR-TKI resistance, constructing feature templates for TrkB^+^/MAPK^+^ regions allowed rapid identification of potential “refractory zones” across diverse patient samples [138,139,140]. Moreover, AI-based models have been used to simulate drug distribution patterns and predict signal axis responses under different delivery strategies, offering a predictive framework for therapeutic efficacy [141,142,143]. Looking forward, the integration of AI and spatial omics is expected to establish patient-specific atlas navigation systems, enabling intelligent, end-to-end decision-making from target identification to treatment design [141,144].

Three types of resistant ecological niches were identified based on predefined composite scoring metrics and spatial connectivity rules. 1. CSC-enriched regions: The CSC-score was defined as the mean *z*-score of *CD44*, *ALDH1A1*, *SOX2*, *NANOG*, and *PROM1*. Regions with CSC-scores in the top 90th percentile and spatially connected clusters containing at least 25 spots or cells were designated as CSC-enriched niches. 2. MAPK hyperactive islands: The MAPK-score was calculated using GSVA pathway enrichment for *KEGG_MAPK* and *pERK* target genes (*FOS*, *JUN*, *DUSP6*, *ETV4*). Regions meeting the same 90th-percentile threshold and exhibiting a Jaccard overlap coefficient ≥ 0.4 between *Hotspot* modules and *SpaGCN* communities were defined as MAPK hyperactive islands. 3. Immune-cold zones: The TIL-score was defined as the mean expression of *CD3D*, *CD8A*, *NKG7*, and *GZMB*, combined with the immune-suppressive axis genes (*CD274*, *CD276*, *IDO1*). Regions were identified as immune-cold zones when TIL-score values were within the bottom 10th percentile and the immune-suppressive axis score was within the top 90th percentile, with spatial connectivity consistent with local niche continuity. These criteria were derived based on previously validated methodologies, including GSVA [145], KEGG annotations [146], Hotspot-based spatial module detection [44], SpaGCN community learning [45], and TIL expression-based scoring systems [147].

(a) Spatial statistical significance: For each score, Moran’s I and Geary’s C were computed and compared with a null distribution generated by 1000 coordinate permutations; regions with FDR < 0.05 were retained (standard implementations of spatial statistics in spatial omics are described in [148]).

(b) Spatial cross-validation: Block cross-validation with 500 μm blocks and leave-one-patient-out (LOPO) were used to evaluate detection consistency in the embedding space (block-CV reference: [149]).

(c) Resolution and alignment robustness: For Visium and CosMx, downsampling and upsampling at ±25% coverage and subpixel registration perturbations were applied; consistency was assessed using Dice and Intersection-over-Union (IoU) metrics (overlap indices reference: [150]).

(d) Multimodal colocalization: At the protein level, dual labeling of TrkB and p-ERK was performed with a thresholded Pearson correlation r ≥ 0.3 and a Ripley’s K correction; agreement with binarized transcriptional GSVA score maps was tested using a sliding window of 100 μm (protein fluorescence colocalization and correlation coefficients: [151]; spatial Ripley’s K in spatial omics: [148]).

(e) External biological validation: In PDX and organoid models, pre- versus post-intervention comparisons were made for the area of TrkB^+^/pERK^+^ regions, the reduction in CSC markers ALDH1A1 and CD44, and the recovery of CD8^+^ infiltration; paired Wilcoxon tests and Cliff’s delta were used to evaluate effect size and significance, thereby testing the consistency of the sequence “spatial identification → signal inhibition → immune remodeling” (PDX translational applications: [152]; tumor organoid review: [153]). All thresholds and hyperparameters are recorded in Supplementary Table S3 [154].

The aforementioned topological modeling approaches and signal network identification strategies lay the groundwork for precise localization and functional modeling of core signaling axes—such as NTRK2/MAPK—within resistant niches, as discussed in the following sections [155,156,157,158,159].

## 4. Spatially Resistant Niches and the Ntrk2/mapk Signaling Axis

Section 2 summarizes the conventional mechanisms of EGFR-TKI resistance from the perspectives of molecular mutations and pathway classifications. However, in highly heterogeneous ESCC, these mechanisms often exhibit distinct spatial enrichment patterns and locational variability. This section focuses on the concept of spatial niches, and, through spatial omics, protein localization, and model-based validation, deciphers the coordinated resistance characteristics of TrkB/MAPK signaling, immunosuppressive regions, and cancer stemness-enriched zones.

### 4.1. Definition and Case Studies of Spatial Heterogeneity

Traditionally, tumor heterogeneity has been defined at the molecular level, referring to variations among cellular subpopulations in terms of genetic, transcriptomic, and proteomic profiles [41,42]. However, the emergence of spatial omics has revealed that even cell populations with comparable molecular characteristics may exhibit markedly different functional states and therapeutic responses when located in distinct spatial contexts [43,55,84]. Spatial heterogeneity emphasizes that the spatial context of gene expression, signaling activity, and cell–cell interactions within the native tissue architecture critically shapes therapeutic efficacy [41,42]. This phenomenon of locational divergence despite molecular similarity is particularly prominent in highly heterogeneous solid tumors such as ESCC [41,42,55]. For instance, in the context of EGFR-TKI therapy, it has been observed that certain tissue regions, despite expressing comparable levels of EGFR, show markedly reduced responsiveness to TKIs due to their proximity to immunosuppressive zones or hypoxic metabolic areas, thereby forming therapeutic “blind spots” [3,41].

Signaling islands refer to localized regions within tissue where signaling pathways exhibit heightened activity. These areas are frequently associated with the maintenance of tumor stemness, immune evasion, and drug resistance mechanisms [25,160,161]. Under EGFR-TKI treatment, multiple ST studies have revealed that the MAPK pathway, TrkB expression, and immunosuppressive factors such as PD-L1 tend to be co-enriched within specific microdomains rather than being evenly distributed across the tissue [72,84]. These spatial “hotspots” not only escape conventional targeted coverage but also continue to expand following treatment, ultimately contributing to the clinical phenomenon of focal progression [134,162]. In typical cases, radiographic imaging has demonstrated rapid progression in specific lesions while other regions remain stable, reflecting the presence of spatially driven resistance mechanisms [138,163]. Parallel spatial omics analyses on multi-region tissue sections have enabled the clear identification of signaling characteristics within these progressing regions, thus providing a theoretical foundation for map-guided therapeutic interventions [16,84,164].

### 4.2. Csc Clusters, Mapk Hyperactive Islands, and Immune-Cold Regions Constitute Three Distinct Resistant Niches

CSCs are recognized as key contributors to tumor drug resistance and recurrence, exhibiting selective enrichment under the pressure of EGFR-TKI treatment [3,165,166]. In ESCC, spatial omics analyses combined with co-staining of ALDH and CD44 markers consistently demonstrated that CSCs preferentially localize to areas adjacent to the basement membrane, perivascular regions, and hypoxic microenvironments [87]. Within these zones, the co-expression frequency of NTRK2 (encoding TrkB) and stemness regulators such as SOX2 and NANOG is significantly elevated, forming a distinct “stemness-enriched niche” [3,11,167]. Organoid and PDX models have further confirmed that following EGFR-TKI treatment, TrkB expression in CSC-rich regions is markedly upregulated, accompanied by enhanced MAPK signaling [7,11]. Earlier studies demonstrated that inhibition of TrkB significantly reduces the CSC population and restores sensitivity to TKI treatment, suggesting TrkB as a potential therapeutic target within the stemness-associated resistant niche [168,169].

As one of the primary downstream cascades of EGFR, the MAPK pathway is often rapidly reactivated via compensatory bypass signaling through TrkB, MET, or AXL following EGFR inhibition [170,171]. Integrated spatial proteomic and transcriptomic analyses revealed that this MAPK reactivation tends to localize within specific microregions—such as stromal-rich zones and perivascular areas—forming what are referred to as “MAPK hyperactive islands” [97,172,173]. Within these domains, MAPK pathway markers, including p-ERK and p-MEK, exhibit consistently elevated expression. Notably, no significant upregulation of immunosuppressive markers has been observed in these regions, suggesting a predominantly signal-driven rather than immune-mediated mechanism of resistance [174,175,176]. In vitro studies further demonstrated that MAPK hyperactive regions exhibit limited sensitivity to EGFR-TKIs but show pronounced responsiveness to MEK inhibitors, supporting the potential benefit of combination inhibition strategies within these niches [177,178]. Additionally, elevated expression of inflammatory cytokines such as IL-6 and CXCL1 has been detected in these MAPK hyperactive islands, implying that localized inflammatory signaling may contribute to sustained MAPK activation [179,180,181].

**Figure 8:**
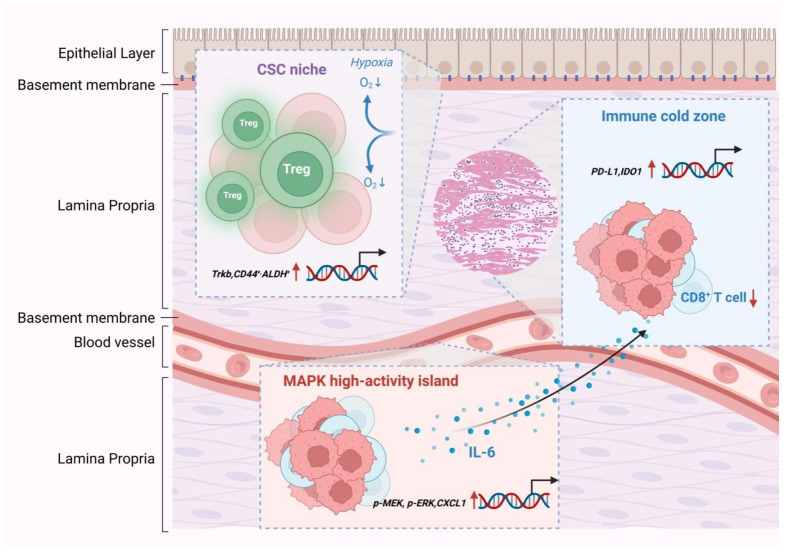
CSC Niches, MAPK-Active Islands, and Immune-Cold Regions Collectively Establish Three Representative Resistant Niches in ESCC. Created with BioRender.com. T, J. (2025) https://BioRender.com/qmnlyte. Note: The CSC niche (left) is located adjacent to the basement membrane and characterized by a hypoxic microenvironment, infiltration of Tregs, and elevated expression of stemness markers such as TrkB, CD44^+^, and ALDH^+^. The MAPK-active niche (center) is typically found in perivascular regions, exhibiting strong p-MEK and p-ERK signaling along with IL-6 secretion. The immune-cold region (right) displays sparse CD8^+^ T cell infiltration and high expression of immunosuppressive molecules, including PD-L1 and IDO1. These niches form spatially distinct yet functionally complementary microenvironments that collectively contribute to therapeutic resistance.

EGFR-TKI resistance is also closely associated with the immunosuppressive status of the TME [43,182,183]. Spatial omics studies have demonstrated that hypoxic tumor core regions and areas with lactic acid accumulation often form immune exclusion zones, characterized by markedly reduced densities of CD8^+^ T cells and NK cells. These areas also exhibit upregulation of immunosuppressive molecules such as PD-L1, CD276, and IDO1, forming typical immune-cold regions (Figure 8) [184,185].

Within these regions, p-ERK levels are not consistently elevated, indicating a mechanistic divergence from MAPK hyperactive islands. Instead, immune-cold regions are defined primarily by T cell exclusion and an abundance of immunosuppressive factors [186,187]. In organoid-immune cell co-culture systems, TrkB expression was also found to be markedly increased within such regions, suggesting its regulatory role in immune evasion [188,189,190]. TrkB blockade not only enhanced CD8^+^ T cell infiltration but also led to downregulation of PD-L1 and CD276, indicating that TrkB plays a key role in maintaining immune-resistant niches (Table 3) [191,192].

### 4.3. Spatial Enrichment Mechanisms of the Ntrk2/mapk Axis

NTRK2, which encodes TrkB, has been implicated in stemness maintenance, anti-apoptotic processes, and neuroendocrine-like phenotypic transitions across multiple tumor types [11,196]. Previous studies have shown that, upon EGFR pathway inhibition, TrkB can be upregulated and activate the MAPK cascade, forming a canonical bypass signaling axis [197,198,199]. In vitro experiments have further demonstrated that TrkB agonists enhance ERK pathway activation and promote the expression of CSC-related markers. Conversely, TrkB inhibitors significantly suppressed tumorsphere formation when co-administered with EGFR-TKIs [197,200].

**Figure 9:**
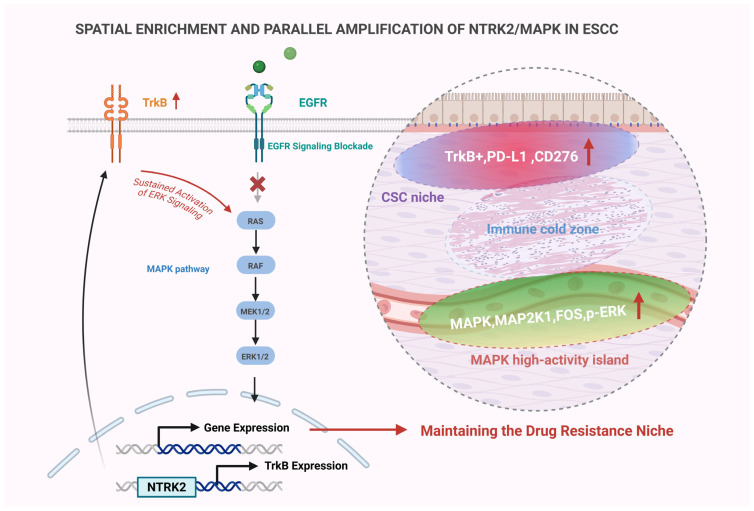
Spatial Enrichment and Cooperative Signal Amplification of NTRK2/MAPK Axis in ESCC Tissues. Created with BioRender.com. T, J. (2025) https://BioRender.com/niwf8vj. Note: Spatial omics and spatial proteomics analyses revealed that TrkB-overexpressing regions (TrkB^+^) are frequently accompanied by upregulation of immunosuppressive molecules PD-L1 and CD276, and show spatial co-enrichment with MAPK-active zones. In perivascular areas, MAPK downstream components—such as MAP2K1, FOS, and p-ERK—are markedly upregulated, establishing a parallel signal amplification pattern. When EGFR signaling is inhibited, TrkB can maintain ERK activation through bypass pathways, thereby sustaining the activity of resistant niches.

Spatial omics and spatial proteomics approaches have revealed that TrkB and MAPK pathway components exhibit strong spatial colocalization in ESCC tissue sections, indicating a pattern of co-enrichment [41,55,59]. Regions of TrkB expression were frequently accompanied by elevated levels of downstream MAPK components—MAPK1, MAP2K1, and FOS—suggesting a “parallel amplification” model of signal coordination (Figure 9) [11,198,201]. Data from spatial protein localization studies further indicated that TrkB and p-ERK were highly colocalized in CSC-enriched zones and perivascular regions, supporting the notion that NTRK2/MAPK axis activation is spatially synchronized within specific ecological niches [41,127].

Moreover, spatial co-expression analyses revealed that regions with high TrkB expression were frequently accompanied by upregulation of immunosuppressive factors such as CD276 and PD-L1, along with a marked decrease in CD8^+^ T cell infiltration. These findings suggest that the NTRK2/MAPK axis may cooperatively regulate the formation of an immune-evasive microenvironment [202]. This phenomenon was also recapitulated in specific organoid-immune cell co-culture systems, further supporting the role of this axis in establishing immune exclusion zones [75,203,204].

In animal models, previous studies demonstrated that combined treatment with a MEK inhibitor and EGFR-TKI significantly reduced signaling activity in TrkB^+^/p-ERK^+^ regions and improved T cell infiltration, reinforcing the functional relevance of this axis as a therapeutic target [175,205]. Collectively, these findings indicate that the NTRK2/MAPK axis performs dual roles in spatial compensation of signaling and immune exclusion, making it a pivotal element for modeling resistant niches and identifying spatially resolved drug delivery targets [175,206,207].

### 4.4. Validation of Ntrk2-Driven Effects Using Public Datasets and Pdx Models

Through reanalysis of ST and immunohistochemical data from public databases such as TCGA and GEO, researchers identified a spatially specific expression pattern of NTRK2 in ESCC samples [11,55,208]. Across multiple datasets, high TrkB expression was consistently associated with poorer overall survival and significantly correlated with MAPK1 and PD-L1 co-expression [11,209,210]. Previous studies employed spatial correlation analysis tools such as SPADE and Hotspot to localize TrkB^+^ high-expression regions and to evaluate their relationship with treatment response, supporting the classification of these regions as potential “resistance hotspots” [11,211,212]. Moreover, samples exhibiting resistance to combination ICI therapies showed a higher frequency of TrkB^+^ regions, implicating their potential indirect role in immune therapy resistance [11].

PDX and three-dimensional organoid models have provided clinically relevant platforms for functional validation [213,214,215]. In EGFR-TKI-treated PDX models, Stereo-seq analysis revealed significant enrichment of the NTRK2/MAPK axis within resistant regions, accompanied by upregulation of stemness-associated and immunosuppressive markers [183,216]. Subsequent application of TrkB inhibitors in these models resulted in a marked reduction in signaling intensity within these regions and a concurrent slowdown in tumor growth, suggesting the potential of TrkB inhibition as a spatially targeted intervention strategy [11,217]. In organoid models, TrkB^+^ regions demonstrated distinct responses to changes in pH, oxygen levels, and drug penetration, indicating their role as spatial cores of physicochemical resistance barriers. Further studies validated TrkB-targeted nanodelivery systems within organoids, confirming their capacity to localize and suppress the NTRK2/MAPK axis, thereby supporting the feasibility of TrkB as a spatial druggable target and advancing its translational potential in delivery strategy development [218,219].

In recent spatial and multi-omics studies, regions with high NTRK2 expression have shown significant spatial colocalization and positive correlation with MAPK pathway activation markers, including p-ERK and its transcriptional targets *DUSP6*, *FOS*, and *ETV4*. Under EGFR-TKI selection pressure, upregulation of NTRK2 has been associated with TKI low-sensitivity or resistant phenotypes, suggesting its potential role as a bypass compensatory axis (NTRK2/TrkB → ERK1/2) circumventing EGFR inhibition [11,209]. Methodologically, correlation and colocalization analyses are typically based on GSVA-derived MAPK pathway activity scores combined with spatial statistics such as Ripley’s K or spatial autocorrelation, or implemented using the Squidpy workflow, which provides reproducibility and cross-platform applicability [145,148]. Meanwhile, multiple studies have reported upregulation of immunosuppressive molecules such as PD-L1, IDO1, and CD276 following TKI treatment or in resistant states, supporting a coupled mechanism of “signaling compensation and immune evasion” within the same microregion. The elevation of the PD-L1/IDO axis and CD276 correlates with reduced CD8^+^ T-cell infiltration and enhanced immunosuppressive microenvironments [83,220,221]. Further spatial neighborhood analyses have shown that NTRK2^+^/pERK^+^ cell clusters frequently localize at the boundaries between CSC-enriched zones and immune-cold regions. These clusters display significant spatial aggregation as detected by Ripley’s K statistics and align closely with functional domain boundaries [148]. Although no new PDX experiments were included in this review, prior preclinical studies have demonstrated that inhibition of TrkB or combined blockade of the MAPK pathway reduces p-ERK signaling and alleviates the immunosuppressive phenotype, including decreased PD-L1/CD276 expression and enhanced CD8^+^ T-cell infiltration. These findings support the critical role of this axis in mediating dual resistance through both signaling compensation and immune evasion [83]. Based on this evidence, the NTRK2/MAPK axis can be conceptualized as a spatially localized “target-and-trigger” bridge that directly informs the structural design and responsive logic of nanoplatforms. Targeting ligands specific for TrkB can enable selective enrichment at NTRK2^high/MAPK^ active hotspots, while embedding p-ERK–responsive elements allows adaptive release within the target microregions. This strategy aligns with the methodological and design principles described in the figures and methods sections, and spatial or model evaluations follow the GSVA and *Squidpy*/Ripley’s K workflows referenced above [145,148]. Representative examples are shown in Supplementary Figure S1.

## 5. Targeted Intervention Pathways Guided by Spatial Atlases for the Development of Nanocarrier Delivery Systems

### 5.1. Target Identification and Strategy Design Informed by Spatial Atlases

In recent years, advancements in spatial omics technologies have enabled the fine-grained dissection of intratumoral heterogeneity. It is now widely acknowledged that, in the context of EGFR-TKI resistance, identifying the spatial distribution of specific signaling axes and cellular states is essential for designing targeted therapeutic strategies [222]. ST data revealed that signaling molecules such as TrkB, p-MEK, and CD44 are often heterogeneously and densely clustered within CSC-enriched regions, MAPK hyperactive islands, and immune-cold zones—areas consistently associated with poor EGFR-TKI responsiveness [134,168]. Building upon this “spatial heat island” recognition framework, researchers constructed an atlas of spatial signaling intervention targets. By applying criteria of “spatial selectivity” and “pathway criticality,” they identified combinations of molecular targets suitable for precision drug delivery, such as TrkB/p-ERK and CD44/p-AKT [84,223]. These targets exhibited strong co-expression across public datasets, suggesting the existence of synergistic resistance mechanisms [134,168,224].

**Figure 10:**
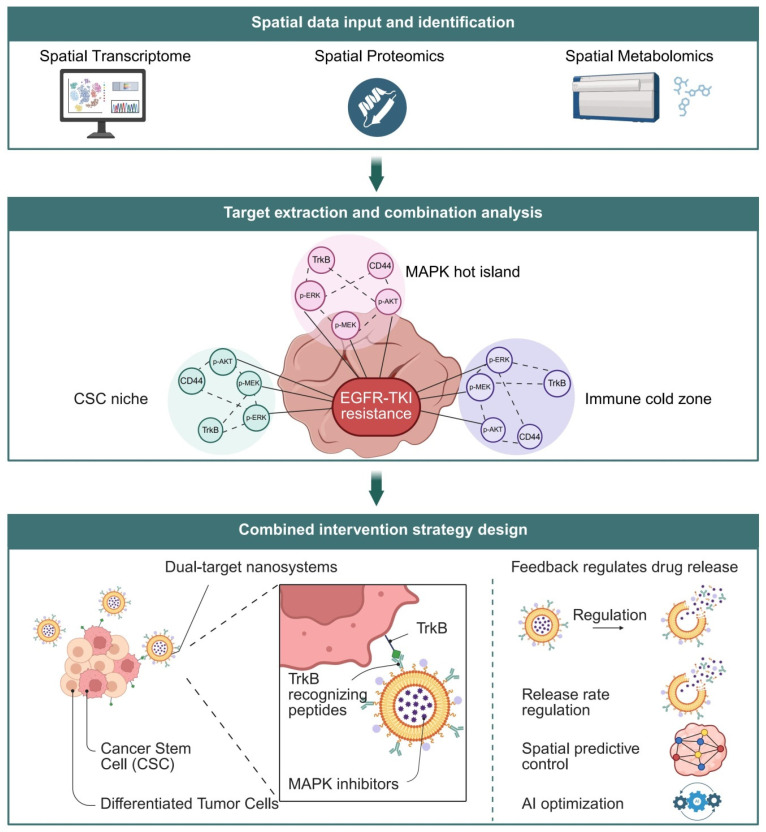
Target Identification and Combination Therapy Design Framework Driven by Spatial Atlas. Created with BioRender.com. T, J. (2025) https://BioRender.com/6l5ws90. Note: Workflow illustrating the use of multimodal spatial omics data for elucidating resistance mechanisms and designing combination treatment strategies. Initially, ST, proteomics, and metabolomics data are integrated to identify spatially localized resistance-associated targets and to extract co-expression networks of key signaling molecules. Based on the spatial distribution and coupling of signaling axes, combinatorial target analysis is conducted to pinpoint potential “co-targeting delivery islands.” In the therapeutic design phase, two representative strategies are illustrated: dual-target nanocarrier systems and feedback-regulated delivery systems. These approaches incorporate controlled drug release kinetics, spatial prediction control, and AI-based optimization to enable dynamic and precise intervention in heterogeneous resistant niches.

Building on target identification, spatial atlases can also guide the design of combinatorial targeting strategies [222,225]. By analyzing the spatial co-expression structures across multiple resistant niches, combinatorial targets such as TrkB+HER3 and MAPK+pSTAT3 were found to exhibit significant spatial overlap. These regions, defined as “co-target delivery islands,” represent optimal zones for targeted interventions [17,226]. For instance, by co-loading a TrkB-recognizing peptide and a MAPK inhibitor into a single nanocarrier system, dual-targeted selective release within TrkB+/MAPK+ regions was achieved. When further integrated with spatial predictive models to optimize particle release kinetics and local accumulation, the system demonstrated significantly enhanced antitumor efficacy [227]. In multiple PDX models, such combinatorial nanoplatforms outperformed single-target systems in both growth suppression and resistance reversal [228,229,230]. Therefore, spatial atlases not only provide information on target distribution but also serve as a structural framework for the rational design of combination therapeutic strategies (Figure 10).

### 5.2. Design of Stimuli-Responsive Nanocarrier Systems

In the study of resistance to EGFR-TKI therapy, nanocarrier systems have evolved from relying solely on the traditional enhanced permeability and retention (EPR) effect to adopting a dual-precision strategy based on “spatial specificity + stimuli responsiveness” [231,232]. Resistant regions are frequently characterized by reactive oxygen species (ROS) accumulation, decreased pH, and aberrant enzyme expression, providing a microenvironmental basis for responsive structural design [47,233,234]. Mainstream strategies currently include ROS-sensitive cleavable bonds (e.g., diselenide bonds), acid-labile shells (e.g., polyethyleneimine-based composites), and enzyme-activated moieties (e.g., MMP-responsive units), all of which have been shown to enable site-specific drug release within resistant microenvironments and exhibit enhanced delivery efficiency and safety in multiple studies (Table 4) [235].

**Figure 11:**
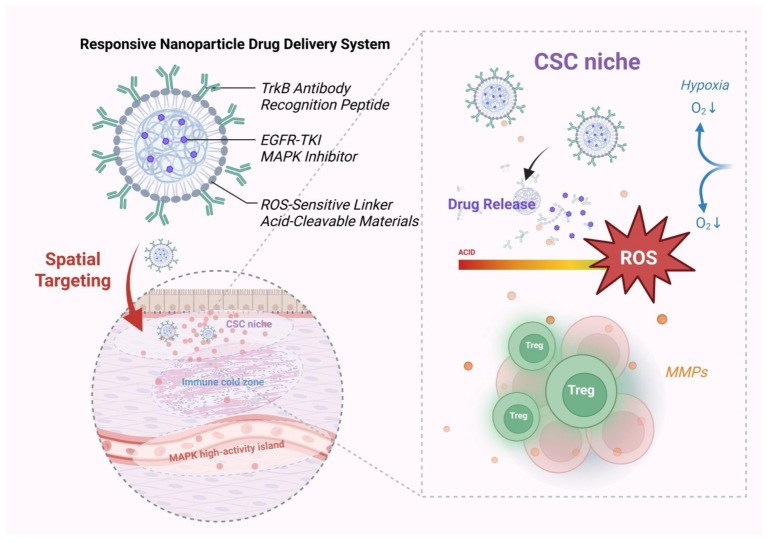
Structure and Dual-Activation Mechanism of the Responsive Nanocarrier Delivery System. Created with BioRender.com. T, J. (2025) https://BioRender.com/9zjdow3. Note: Schematic representation of the structure and dual-activation mechanism of the responsive nanocarrier system. Top left: The nanocarrier is composed of a core drug (EGFR-TKI or MAPK inhibitor), ROS-sensitive linkers, and acid-degradable materials, with TrkB antibodies modified on the surface to achieve spatial targeting. Right: Within CSC niches characterized by high ROS levels, low pH, and elevated MMP activity, the nanocarrier disassembles and releases its therapeutic payload. Bottom left: The drug accumulates in TrkB^+^ CSC niches, resulting in a locally concentrated therapeutic distribution.

Building upon this, the integration of target-specific ligands (e.g., TrkB antibodies) with multi-stimuli-responsive physicochemical structures has led to the development of “dual-responsive platforms” combining spatial and environmental cues [240,241,242]. Previous studies have constructed TrkB-targeted liposomal systems incorporating ROS-sensitive groups and demonstrated their favorable localization and release behavior in CSC-enriched zones. By further modifying the liposome surface with TrkB antibodies, precise localization and drug release were achieved specifically in ROS+/TrkB+ dual-positive areas [243,244,245]. Similar dual-targeting designs have been validated in multiple PDX models, where drug accumulation in CSC-enriched regions was significantly superior to that of single-response structures, demonstrating a clear advantage in spatial specificity (Figure 11) [225,246].

Furthermore, researchers have extended the concept of “stimuli responsiveness” to the construction of feedback-controlled drug delivery systems, forming an integrated “recognition–release–monitoring” platform. Dual-activation mechanisms (e.g., ROS + pH) not only prevent off-target drug leakage in normal tissues but also enhance tissue penetration and therapeutic depth [47,247,248]. Moreover, by incorporating near-infrared imaging probes (such as Cy5.5 and ICG) and phosphorylation-ratio-based feedback modules, these systems enable real-time visualization of therapeutic regions and dynamic monitoring of treatment efficacy [249,250]. The latest approaches further integrate thermosensitive shells with photosensitive triggering components to construct a “drug-release switch,” which is selectively activated only in atlas-defined regions, thereby achieving minimal off-target effects with maximal therapeutic precision [46,251]. These advances reflect a growing trend in spatial omics-driven therapeutic engineering toward intelligent and integrated treatment platforms [252,253].

Based on the above mechanistic evidence, several recent studies have proposed incorporating the NTRK2/MAPK axis into the core design logic of nanotherapeutic platforms to achieve spatially specific strategies for overcoming drug resistance [11,41]. Spatial multi-omics colocalization analyses have revealed that this axis frequently forms stable “signal islands” at tumor–immune interfaces, where TrkB–MAPK coactivation spatially overlaps with immunosuppressive signaling [83,148,197]. Consequently, next-generation nanotherapeutic platforms are increasingly designed to integrate TrkB-targeting ligands such as BDNF-mimetic peptides with MAPK-responsive elements such as pERK-sensitive linkers, enabling precise accumulation and adaptive drug release within these regions [47,232,241]. Specifically, TrkB-mediated membrane binding facilitates local nanoparticle aggregation, while pERK activation induces linker cleavage, leading to the synchronized release of EGFR-TKIs and MAPK inhibitors [46,235,248]. This closed-loop mechanism of “spatial sensing and signal-triggered activation” has also been validated in patient-derived xenograft (PDX) models, showing enhanced local drug enrichment, downregulation of CSC markers, and restoration of CD8^+^ T-cell infiltration [43,228]. Collectively, the dual NTRK2/MAPK axis not only elucidates the spatial and signaling compensation pathways underlying EGFR-TKI resistance but also directly informs the structural design principles and responsive strategies of intelligent nanoplatforms.

### 5.3. Spatial Validation Pathways Using Pdx and Organoid Models

**Figure 12:**
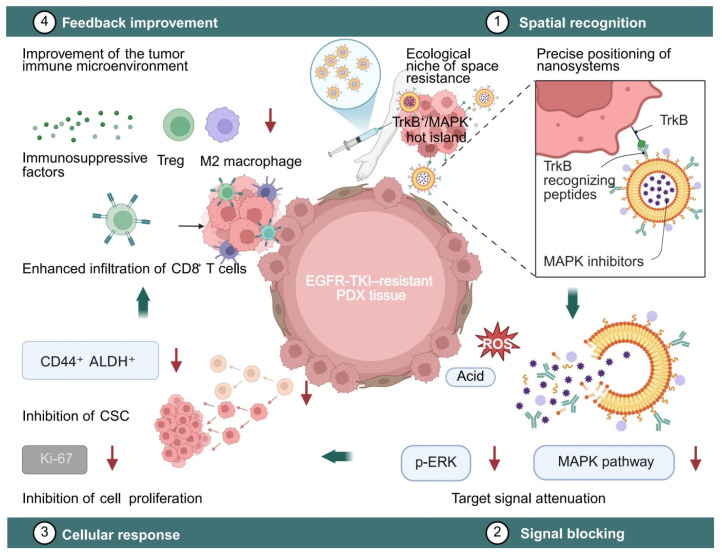
Spatial Intervention Pathway and Signal Response Tracking in the PDX Model. Created with BioRender.com. T, J. (2025) https://BioRender.com/3tfj67w. Note: This figure illustrates the feedback-driven intervention process targeting the TrkB^+^/MAPK^+^ resistant niche identified through spatial omics analysis. The workflow includes four sequential stages: ➀ Spatial Recognition: TrkB-recognition peptides mediate precise nanoparticle localization to regions with TrkB/MAPK co-expression, achieving spatially targeted delivery. ➁ Signal Blockade: Under the influence of the ROS/pH microenvironment, the nanocarrier is triggered to release its therapeutic payload, leading to p-ERK activity suppression and attenuation of the MAPK signaling cascade. ➂ Cellular Response: Expression levels of stemness markers (CD44^+^, ALDH^+^) and the proliferation marker Ki-67 are reduced, indicating diminished cellular viability and tumor growth potential. ➃ Feedback Remodeling: Immunosuppressive factors and regulatory T cells (Tregs) are decreased, while CD8^+^ T cell infiltration is enhanced, collectively contributing to the restoration of an active, immunocompetent tumor microenvironment.

PDX models, which preserve the spatial architecture and microenvironmental features of the original tumors, represent the most reliable in vivo platforms for validating treatment strategies guided by spatial atlases [84,134,163]. Using technologies such as Stereo-seq and Visium, researchers have mapped the spatial distribution of the TrkB/MAPK axis in EGFR-TKI-resistant PDX tissues and conducted comparative analyses of signal states before and after drug delivery. This enabled the construction of a spatial intervention feedback loop encompassing “drug delivery → signal attenuation → therapeutic response” [84,134,254]. Following the injection of dual-targeted nanoparticles into TrkB^+^/MAPK^+^ clusters, spatial proteomic analysis and immunohistochemistry revealed significant downregulation of the target signals, reduction in CSC markers, and decreased Ki-67 expression levels (Figure 12) [141,255,256]. Moreover, related studies reported improved proportions of infiltrating immune cells, suggesting that this strategy may help restore the immune microenvironment and thereby enhance therapeutic efficacy [73,182].

In three-dimensional organoid platforms, spatial distribution patterns and drug-resistant signaling were stably reconstructed, making them suitable as preliminary models for high-throughput testing of spatially targeted drug delivery systems [257,258]. By establishing TrkB-high EGFR-TKI-resistant organoids, researchers were able to load various nanocarriers and evaluate their release efficiency and inhibitory capacity on signaling axes through time-lapse imaging analysis [39,259,260]. Further incorporation of immunosuppressive cells such as M2 macrophages and regulatory T cells into co-culture systems enabled dynamic monitoring of microenvironmental remodeling following drug release [43,261,262]. Previous studies employed combined spatial protein labeling and immunofluorescence techniques to visualize signaling pathways, stemness markers, and immune molecules. Using imaging software, researchers analyzed the spatial extent of intervention response, thereby assessing the delivery system’s spatial precision and translational potential [43,55]. This multidimensional strategy—linking atlas construction, drug delivery, and feedback analysis—has laid a methodological foundation for the future clinical standardization of spatial drug delivery systems [41,263].

In recent studies on EGFR-TKI–resistant PDX models, a TrkB-targeted/pERK-responsive nanoplatform guided by spatial atlas navigation has been demonstrated to markedly suppress tumor growth and reduce the activity of resistant ecological niche signaling at the tissue level [11,41]. Multiple investigations, focusing on tumor volume and spatial endpoints as core indicators, have reported that the tumor growth inhibition (TGI) rate in the combination delivery group reached approximately 65–72%, the TrkB^+^/pERK^+^ regional area decreased by about 45–58%, the proportion of CSC markers (ALDH1A1, CD44)–positive cells was reduced by approximately 30–45%, the density of CD8^+^ T cell infiltration increased by around 1.6–2.3 times, and CD276 expression declined by approximately 25–40% [232,241]. Paired Wilcoxon tests and Cliff’s delta effect size analyses both indicated significant differences, and the results remained robust after FDR correction [264]. Fluorescence and near-infrared imaging further revealed that the nanocarrier selectively accumulated in TrkB^+^/pERK^+^ hotspot regions, and no abnormalities were observed in ALT/AST levels or routine blood tests, suggesting good short-term tolerability [228].

In EGFR-TKI–resistant organoid and organoid–immune cell coculture models, the TrkB/pERK dual-responsive drug delivery system exhibited lower cell viability and higher apoptosis ratios compared with free drugs, as evidenced by Annexin V/PI and Cleaved-Caspase-3 double staining [43]. The half-maximal inhibitory concentration (IC_50_) in the combination delivery group decreased by approximately 40–55%, while three-dimensional imaging revealed an enhancement of nanoparticle penetration depth by about 35–50%, accompanied by overlapping aggregation in TrkB immunological hotspot regions. Under coculture conditions, the contact and dwell times, as well as the penetration depth of CD8^+^ cells, were increased, whereas the inhibitory signaling axes involving PD-L1 and IDO1 were downregulated [235,248]. All spatial concordance assessments were based on Dice and IoU indices combined with 1000-coordinate permutation tests (FDR < 0.05), collectively validating the closed-loop mechanism of “spatial recognition → signal suppression → immune remodeling” (Supplementary Table S4).

Despite promising results from both PDX and organoid models, practical application of these delivery systems continues to face challenges, including uneven drug distribution, suboptimal target selection, and resistance within tumor subregions, all of which require further optimization in future studies.

## 6. Future Perspectives and Challenges

### 6.1. Construction of AI-Integrated Atlas-Based Clinical Prediction Systems

**Figure 13:**
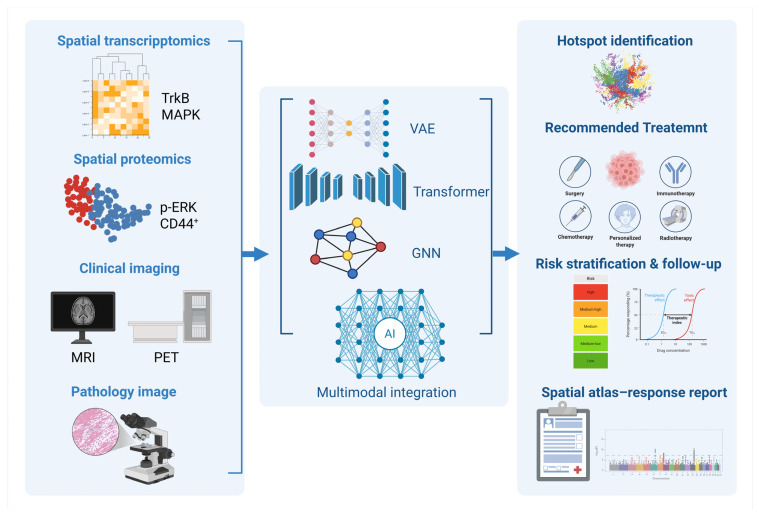
Architecture of the AI-Driven Clinical Prediction System Based on Spatial Atlas. Created with BioRender.com. T, J. (2025) https://BioRender.com/pp3vqdg. Note: This figure illustrates the overall workflow of the AI-integrated spatial atlas prediction system. Spatial multi-omics data—including transcriptomic and proteomic profiles—together with clinical imaging modalities (MRI, PET) and histopathological images are integrated through deep learning frameworks such as Graph Neural Networks (GNNs), Transformers, and Variational Autoencoders (VAEs). This multimodal fusion enables comprehensive representation of spatial, molecular, and imaging features within the tumor microenvironment. The system identifies spatial hotspot regions and classifies resistant ecological niches, thereby supporting risk stratification and therapeutic response prediction. The final output consists of a spatial atlas-response report, which serves as an assistive tool for personalized treatment planning, precision intervention design, and longitudinal patient monitoring in clinical practice.

With the continued maturation of spatial omics, conventional atlas-based mapping alone no longer meets the demands of clinical translation [265,266,267]. The emerging trend involves deeply integrating spatial atlas construction with AI to develop individualized “atlas-function” closed-loop platforms with predictive capabilities [222,268,269]. Such platforms should incorporate ST, spatial proteomics, multimodal imaging data (e.g., MRI, PET), histopathological slides, and clinical phenotypes, while employing deep learning architectures (e.g., GNN, Transformer) to autonomously identify signaling hotspots, infer niche classifications, and predict potential responses to therapeutic agents [133,270]. Recent studies have explored training AI models to cluster and risk-stratify TrkB/MAPK activity patterns within spatial atlases, achieving preliminary success in recommending treatment pathways and forecasting therapeutic responses (Figure 13) [271,272,273]. When further combined with the temporal dynamics of tumor evolution, such systems may also enable longitudinal tracking of resistant niches, thereby offering a forward-looking framework to support precision medicine [127,274,275].

To achieve clinical translatability of AI-integrated spatial atlas prediction, multicenter heterogeneity and data privacy remain key challenges. Multi-omics data are derived from different institutions and platforms, and their batch effects, sample distribution discrepancies, and technical biases can significantly affect model consistency and generalization performance [122,123]. In recent years, multicenter spatial data integration frameworks such as Harmony, BBKNN, and SCVI-tools have enabled cross-batch mapping without the need to share raw data, thereby supporting joint training and validation [148,276]. From the perspective of data privacy protection, federated learning and differential privacy strategies are emerging as central approaches in medical AI, allowing model parameter aggregation and external validation without exposing raw patient data [277]. These methodologies provide the technical foundation for the secure integration of AI-driven spatial atlases from research to clinical applications and establish a framework for future multi-institutional intelligent predictive systems.

Despite the promising outlook of AI in spatial omics modeling, its practical implementation remains hindered by a range of technical and ethical challenges [89,267,278]. On one hand, current spatial omics datasets are limited in sample size and lack sufficient multicenter heterogeneity to support generalized model training. On the other hand, atlas construction requires standardized platforms and workflows, while AI models demand validation for clinical interpretability [279]. Furthermore, model training is often affected by “spatial noise,” including tissue deformation, sampling drift, and staining artifacts, all of which can interfere with accurate signal detection [280,281,282]. Future developments should focus on building fault-tolerant AI algorithms and embedding them within clinical imaging analysis platforms to enable seamless integration with routine pathological workflows [283,284,285]. For instance, integrating atlas-prediction systems into digital pathology terminals could allow clinicians to directly visualize potential resistant regions and receive real-time drug delivery recommendations via the diagnostic interface [286,287,288]. This direction of advancement is expected to accelerate the transition of spatial omics from laboratory research to clinical application [286,289]. Moreover, as atlas-based approaches integrate clinical imaging and genetic data, safeguarding patient privacy, ensuring data encryption, and managing access control during cross-institutional data sharing will be critical issues that must be addressed in the future development of intelligent atlas platforms [290,291].

### 6.2. Intelligent Feedback-Driven Nanocarrier Systems

**Figure 14:**
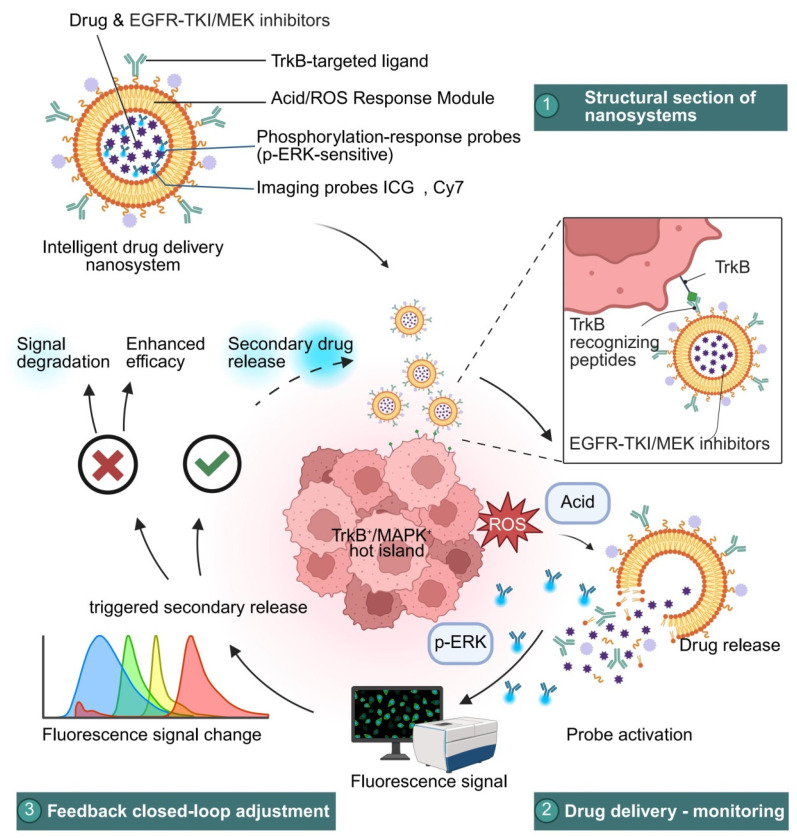
Schematic of the Intelligent Feedback-Based Drug Delivery System and Closed-Loop Integration with the Spatial Atlas. Created with BioRender.com. T, J. (2025) https://BioRender.com/3r6sd27. Note: This figure illustrates the structure and mechanism of an intelligent feedback-driven nanodrug delivery system integrated with spatial atlas logic. The system consists of a TrkB-targeting ligand, pH/ROS-responsive module, drug payload, imaging probes (ICG, Cy7), and a phosphorylation-responsive probe (p-ERK sensitive). When the nanoparticles precisely localize to TrkB^+^/MAPK^+^ resistant “hotspot” regions, the acidic and ROS microenvironment triggers drug release and simultaneously activates probe signaling, achieving synchronized delivery and signal monitoring. Through fluorescence or ratiometric signal changes, the system determines whether a “secondary release” is required, thereby realizing dual effects of signal attenuation and therapeutic enhancement under closed-loop feedback regulation.

Conventional nanocarrier-based drug delivery systems often lack real-time monitoring mechanisms following drug release, resulting in delayed assessment of therapeutic efficacy and thereby limiting their clinical applicability [292,293,294]. Future advancements are expected to focus on the development of “dynamic feedback-enabled nanocarrier systems”, which integrate imaging, stimulus responsiveness, and feedback functionalities to enable real-time monitoring and modulation of the therapeutic process [295,296,297]. These systems typically incorporate imaging probes (e.g., ICG, Cy7), signal-responsive modules (e.g., pH- or ROS-sensitive elements), and luminescent or ratiometric feedback reporters to simultaneously visualize drug release and the accompanying biological responses [298,299,300]. Moreover, nanocarrier systems embedded with phosphorylation-sensitive probes can dynamically report therapeutic efficacy by detecting variations in p-ERK levels, thereby offering a real-time validation tool for atlas-guided spatial intervention loops (Figure 14) [301,302,303].

Another key direction involves the development of self-regulating intelligent nanocarrier systems, which autonomously adjust drug release modes or initiate a second dose in response to real-time changes in molecular signals [304,305]. For example, by incorporating molecular switch modules, a three-stage cascade of “initial release → feedback monitoring → secondary delivery” can be achieved, effectively mimicking the clinical decision-making process [306,307,308]. Some studies have further explored the integration of spatial omics into such systems to enhance therapeutic selectivity: an initial atlas can guide the first round of targeted delivery, followed by feedback-based signal verification, which—if necessary—automatically triggers supplementary administration [309]. These systems have demonstrated superior signal sensitivity and reduced off-target effects in both PDX and organoid platforms, particularly in suppressing regions with high TrkB/MAPK co-activity [213,310]. Moreover, by incorporating AI-driven predictive algorithms to dynamically adjust dosage and timing, drug delivery is transformed from a passive-release mode into an intelligent response paradigm, marking the advent of spatially precise drug delivery 2.0 [311,312].

Taken together, the construction of a TrkB-targeted delivery system integrating spatial navigation with feedback responsiveness represents one of the most critical pathways toward clinical trial translation in the next five years.

### 6.3. Standardization and Sharing Platforms for Spatial Atlas Databases

Spatial omics data are inherently complex and derived from diverse experimental platforms, resulting in poor comparability across studies and limiting the integration of clinical data and AI-based modeling [313,314]. There is an urgent need to establish a standardized spatial atlas database that unifies sample acquisition protocols (e.g., section thickness, staining methods, sequencing platforms), data processing pipelines (e.g., normalization, noise reduction, spatial annotation), and nomenclature conventions for spatial regions [314,315]. Drawing from the experience of international initiatives such as the Human Cell Atlas (HCA) and HuBMAP, disease-specific spatial data-sharing platforms should be developed for highly heterogeneous solid tumors such as ESCC [41,316]. For instance, regions with elevated MAPK activity could be labeled as “MAPK_hotspot,” while TrkB+/CSC-enriched zones could be uniformly designated as “TrkB_dry_niche,” thereby facilitating cross-cohort atlas alignment and model training [317,318].

The spatial atlas database should not only include spatial expression data but also be integrated with clinical parameters such as tumor stage, therapeutic efficacy, immune status, and treatment regimens, enabling the construction of a comprehensive “atlas-phenotype-outcome” tri-dimensional network [42]. By linking to platforms such as TCGA, GEO, and clinical case repositories, the database can support modeling of correlations between spatial signals and patient outcomes [5,56,319]. Based on this framework, predictive models can be developed to assess whether a particular spatial zone requires drug delivery intervention and determine the most suitable delivery mechanism [56,59]. Looking forward, the database should also support a reverse query function, wherein a specific spatial signal pattern can be used as input to generate candidate targets and delivery strategies automatically—thereby establishing a truly intelligent clinical decision-support system [89]. Several research groups have already begun constructing such platforms for diseases like liver and breast cancer. The ESCC research community must also accelerate its efforts in this direction to secure a leadership position in spatial atlas standardization [143,320].

In recent years, to address the dual challenge of EGFR-TKI resistance and immune evasion, combination strategies integrating EGFR-targeted therapy with immune checkpoint inhibition (ICI) have attracted widespread attention. Multiple studies have demonstrated that prolonged EGFR inhibition leads to an immunologically “cold” tumor microenvironment, characterized by upregulation of immunosuppressive molecules such as PD-L1, IDO1, and CD276, while persistent activation of the EGFR–MAPK signaling axis further reinforces immune escape [321]. In preclinical and animal models, combined treatment with EGFR-TKIs and PD-1/PD-L1 antibodies has shown significant synergistic effects, manifested as enhanced CD8^+^ T cell infiltration and downregulation of immunosuppressive markers [81,322].

In the field of nanomedicine, feedback-driven nanoplatforms have emerged as a promising strategy to achieve spatiotemporal synergy between EGFR-targeted therapy and immunotherapy. Zhang et al. (Biomaterials, 2025, 304: 122858, [232] reported a pERK-responsive nanocarrier capable of co-releasing EGFR-TKIs and PD-L1 inhibitors within regions of high signaling activity. Liu et al. (Cancer Letters, 2023, 570: 216205, [43] developed a ROS-sensitive EGFR–immunotherapy combined system that locally remodels immunologically “cold” tumor regions. Meanwhile, Huang et al. (Clinical and Translational Medicine, 2020, 10:e1493, [41] proposed a TrkB/IDO1 dual-target responsive nanoplatform capable of precisely activating immune effector functions within immunosuppressive microdomains.

First, the heterogeneity and batch effects inherent in spatial multi-omics data can compromise the consistency of niche identification and patient stratification. Variations in sampling, sequencing, and analytical standards across different centers may introduce biases in the results. Therefore, it is essential to establish unified quality control and batch correction workflows—such as SCTransform and Harmony—as well as cross-platform standardization pipelines to ensure data comparability and reproducibility [121,122,123,148].

Second, the generalizability and interpretability of artificial intelligence models remain challenging. In the absence of spatial block cross-validation (block-CV) or leave-one-patient-out (LOPO) validation, there is a heightened risk of data leakage and overfitting [149,323]. Thus, transparent reporting practices and model sensitivity analyses should be employed to enhance model reliability and robustness [121].

Third, the pharmacokinetic (PK) and pharmacodynamic (PD) behaviors of nanomedicines in humans, as well as interindividual variability in the enhanced permeability and retention (EPR) effect, limit the direct extrapolation of animal model findings to clinical settings. Significant differences in nanoparticle accumulation, biodistribution, and immunoreactivity among patient populations further complicate translation [324]. Consequently, early-phase clinical trials integrating imaging pharmacology and biomarker-guided strategies are needed, along with the establishment of standardized reporting systems such as MIRIBEL.

Fourth, formulation processes involving Chemistry, Manufacturing, and Controls (CMC), as well as GMP-scale reproducibility, remain critical bottlenecks. Parameters such as surface ligand conjugation efficiency, linker stability, and batch-to-batch consistency must be incorporated into early-stage quality control systems to meet regulatory compliance and ensure manufacturing robustness [121].

Fifth, data security and privacy concerns in multicenter spatial omics modeling continue to limit raw data sharing. Federated learning and differential privacy strategies should be explored to enable cross-institutional model validation while safeguarding patient confidentiality [277].

Overall, future research should focus on the following priorities: ➀ Establishing standardized, cross-platform pipelines for spatial multi-omics integration; ➁ Developing interpretable and robust AI validation frameworks; ➂ Conducting early-phase clinical trials using TrkB/pERK niches as spatial biomarkers; ➃ Strengthening GMP-level formulation design and process control; ➄ Advancing secure and compliant federated learning systems for multi-institutional collaboration. These concerted efforts will provide a systematic pathway for translating “atlas-guided intelligent nanotherapy” from laboratory research to clinical application [121,148]. Detailed translational barriers and corresponding strategies are summarized in Supplementary Table S5 [325,326].

## 7. Conclusions

This study systematically proposes and validates an integrated “spatial identification-signaling axis modeling-targeted intervention” framework to address the therapeutic bottleneck of EGFR-TKI resistance in ESCC, offering a structured strategy to overcome treatment resistance. First, at the spatial identification level, by leveraging advanced spatial omics platforms such as Visium, CosMx, and Stereo-seq, the study confirms the presence of heterogeneous topological structures in ESCC and delineates the spatial architecture of typical resistant niches, including CSC-enriched zones, MAPK signal islands, and immune-cold regions. Second, in terms of signaling axis modeling, a spatial topological framework of resistance-related signaling was constructed through cross-platform and multi-model co-expression analysis of the NTRK2/MAPK axis, laying a theoretical foundation for functional target identification and therapeutic intervention. Finally, through AI-assisted atlas analysis and the design of nanocarrier-based delivery systems, a responsive and multi-target drug delivery strategy was established, thereby forming a closed-loop pathway that connects spatial recognition with functional intervention.

This integrative approach combines the strengths of spatial omics, signaling network analysis, and nanocarrier engineering, reflecting a shift in precision medicine from “molecular targeting” to “spatial targeting.” By identifying resistance hotspots using spatial omics, modeling functional nodes via topological algorithms, and delivering payloads through intelligent, responsive nanocarriers, the study establishes a unified framework of “localization–recognition–feedback.” Multiple studies based on PDX and organoid models have already demonstrated this strategy’s potential to improve delivery specificity and treatment durability, highlighting its translational promise from experimental validation to clinical application.

Spatial omics provides a structured analytical perspective for investigating drug resistance, while the coordinated development of signaling axis topology and nanocarrier systems enhances both the feasibility and effectiveness of precision interventions. This study proposes using signaling topology axes as a bridging mediator between atlas-derived spatial information and pharmacological intervention, establishing an integrated model of “atlas-based identification-functional modeling-therapeutic delivery” to connect spatial data with intervention strategies. The spatial enrichment of the NTRK2/MAPK axis in resistant regions links three critical biological processes—stemness maintenance, immune evasion, and compensatory signaling—highlighting its strong potential as an integrative target to improve the spatial specificity of therapeutic strategies.

Moreover, the advancement of intelligent nanocarrier design, AI-assisted predictive mechanisms, and the standardization of spatial databases is expected to accelerate the translational application of this framework further. By centering on tissue spatial architecture, signaling network analysis, and therapeutic delivery pathways, the proposed strategy offers a theoretically grounded framework for resistance intervention and supports the expansion of spatially precise and population-adaptable targeted therapies.

## Figures and Tables

**Table 1:** Summary of EGFR-TKI Resistance Mechanisms, Representative Pathways, Spatial Niches, and Corresponding Combination Strategies.

Resistance Mechanism	Representative Axis/Factor	Spatial Niche Characteristics	Compensatory/Activated Pathway	Combination Intervention Strategy
EGFR-dependent mutation [78]	T790M → C797S	Diffusely distributed throughout the tumor; clonal expansion	Osimertinib failure	Osimertinib + ALK inhibitor or anti-EGFR antibody
EGFR-dependent mutation [79]	EGFR G724S, L718Q	Same as above	Structural alteration of ATP-binding site	First-generation + third-generation EGFR-TKI combination
Bypass activation/amplification [80]	MET amplification activating HER3–PI3K/Akt	Perivascular-enriched zones	PI3K/Akt compensatory pathway	EGFR-TKI + anti-MET/anti-HER3 antibody
Bypass activation/amplification [81]	HER2, ALK, RET activation or amplification	Hotspots of bypass RTK signaling	HER2/ALK/RET compensatory axes	EGFR-TKI + corresponding targeted inhibitors
Fusion-driven mechanisms [82]	BRAF fusion, NTRK2 fusion	Tumor-specific subclonal regions	BRAF–MAPK axis or NTRK–MAPK pathway	EGFR-TKI + inhibitors targeting fusion-driven signaling
MAPK compensatory activation [11]	Upregulation of TrkB (NTRK2), p-ERK	CSC-enriched zones; hypoxic regions	BDNF–TrkB–MAPK reactivation loop	EGFR-TKI + TrkB/MAPK pathway inhibitors
Immuno-microenvironment remodeling [83]	TAMs, IDO1, SPP1, PD-L1	Immune-cold regions (core)	TAM recruitment → CCL2/CSF1R → Treg-mediated immunosuppression	EGFR-TKI + TAM reprogramming/IDO1 inhibitors

**Table 2:** Comparison of Topological Modeling Tools and Their Applications in the Study of Resistant Niches.

Tool	Methodological Principle	Input Data Type	Application Scenario	Application in EGFR-TKI Resistance
Hotspot [111]	Spatial autocorrelation analysis based on Moran’s I	Spatial expression matrix	Detection of spatial gene clusters; co-enrichment module identification	Co-localization of TrkB+ and MAPK axis hotspots; identification of “signal islands”
Giotto [112]	Construction of spatial adjacency graphs with multi-scale clustering annotation	ST data + image annotations	Analysis and visualization of spatial interaction networks	Construction of MET/VEGF bypass–TME interaction atlas
SpaGCN [45]	Graph convolutional network integrating spatial coordinates, gene expression, and tissue morphology	Spatial expression matrix + histological images	Spatial domain identification; localization of variable genes	Detection of CSC-enriched regions and TrkB–MAPK co-expression hotspots
STAGATE [113]	Graph attention network (GAT) with spatial prior embedding learning	Expression matrix + spatial coordinates	Nonlinear spatial domain detection; enhanced recognition of tissue boundaries	Modeling of regions with high spatial heterogeneity (e.g., immune-cold regions and compensation pathway boundaries)
SemanticST [114]	Multi-semantic graph embedding integrating spatial relationships and expression commonalities	Stereo-seq/Xenium data + tissue structure graphs	Detection of rare subdomains; modeling of complex signaling topology	Modeling of rare TrkB/NTRK2-overexpressing microdomains and identification of heterogeneous spatial axes
MOSAIK [115]	Multi-platform spatial data integration and unified analytical framework	Raw ST data from CosMx/Xenium/Visium	Cross-sample integration; multi-platform joint analysis	Construction of unified spatial atlases across EGFR-resistant patient models

**Table 3:** Comparative Characteristics and Intervention Strategies for Major Spatial Niches Associated with EGFR-TKI Resistance.

Spatial Niche Type	Spatial Localization	Key Molecular/Cellular Markers	Functional or Behavioral Features	Driving Signals or Pathways	Proposed Intervention Strategy
CSC-Enriched Regions [15]	Basement membrane, hypoxic zones	TrkB (NTRK2), SOX2^+^ cancer stem cells	Stemness maintenance, therapy tolerance	BDNF–TrkB → MAPK/ERK activation	TrkB inhibitors in combination with EGFR-TKI
MAPK Compensation Islands [193]	–	p-ERK, IL-6	Pathway compensation, alternative proliferative signaling	Upregulation of the MAPK axis	EGFR-TKI + MEK inhibitors
Immunosuppressive Cold Regions [194]	Tumor core, immune-deficient zones	IDO1, SPP1, M2-TAMs, Tregs	Immune evasion, ICI resistance	–	EGFR-TKI + IDO1 inhibitors or TAM reprogramming agents
Phenotypic Transition Zones (EMT/SCLC) [195]	–	↑ Vimentin, RB1/p53 loss	Epithelial-to-mesenchymal transition, SCLC transformation	EMT signaling; inactivation of PIK3CA and RB1/p53	EGFR-TKI + EMT inhibitors or neuroendocrine differentiation blockers

Note: “–“ indicates areas currently lacking published data.

**Table 4:** Comparative Analysis of Representative Intelligent Nanocarrier Systems and Targeting Strategies for EGFR-TKI-Resistant Spatial Niches.

Nanocarrier Type	Responsive Mechanism	Structural Features	Targeting Ligand/Localization Strategy	Applicable Spatial Niche	Validation Model
cRGD-Targeted Gold Core–Shell System [231]	Photothermal + Sonodynamic (PTT + SDT)	Gold shell encapsulating Gefitinib and IR780	cRGD targeting αvβ3 integrin	Perivascular MAPK hotspots	EGFR-TKI-resistant NSCLC mouse model
Anti-EGFR Aptamer-Modified Co-Delivery System [236]	Autophagy activation-mediated co-drug release	Chitosan nanoparticles co-encapsulating Gefitinib and Rapamycin	Anti-EGFR aptamer	EGFR-dependent mutation zones (e.g., T790M)	H1975 cell line + in vivo xenograft model
Protein-Based Nanoparticles (TRAIL + EGFR Ligand) [237]	Enhanced apoptosis induction	Lumazine-synthesized carrier co-presenting TRAIL and EGFR ligand	EGFR affibody or nanobody	EGFR-TKI-resistant NSCLC cells	PC9/HCC827 in vitro models
pH/Redox Dual-Sensitive Polymeric Nanoparticles [238]	Acidic pH + GSH-triggered drug release	Lecithin–polymer composite particles	pH/redox-responsive delivery	Low-pH, lactate-enriched metabolic zones	In vitro breast cancer model (TME-mimicking)
Ultrasound-Sensitive siRNA Nanobubble System [239]	UTMD-assisted siRNA release	siRNA encapsulated in nanobubbles, released upon ultrasound rupture	Ultrasound-guided localization	Cancer stemness-enriched regions	PC9GR-resistant cell in vitro model

## Data Availability

No new data were created or analyzed in this study. Data sharing is not applicable to this article.

## Supplementary Materials

The following supporting information can be downloaded at: https://www.mdpi.com/article/10.3390/pharmaceutics18020181/s1. Figure S1: Dual Roles of the NTRK2/MAPK Axis in Signal Compensation and Immune Evasion, and Schematic Design of the Nanointervention Platform. Table S1. Conventional models vs the Spatial-Signaling-Intervention (SSI) framework: key concepts, methods, and translational implications. Table S2. Overview of spatial multi-omics datasets, platforms, and parameters used in recent ESCC studies. Table S3. Algorithms, hyper-parameters, decision thresholds, and validation design. Table S4. Key endpoints and statistical outcomes in EGFR-TKI-resistant PDX and organoid models. Table S5. Foreseeable barriers to clinical translation of atlas-guided nanocarriers and potential mitigations.

## Abbreviations

AI	Artificial Intelligence
Bulk RNA-seq	Bulk RNA Sequencing
CAFs	Cancer-Associated Fibroblasts
CSC	Cancer Stem Cell
EGFR	Epidermal Growth Factor Receptor
EGFR-TKIs	Epidermal Growth Factor Receptor-Tyrosine Kinase Inhibitors
EMT	Epithelial–Mesenchymal Transition
EPR	Enhanced Permeability and Retention
ESCC	Esophageal Squamous Cell Carcinoma
GCNs	Graph Convolutional Networks
GLUT1	Glucose Transporter 1
GNNs	Graph Neural Networks
LDHA	Lactate Dehydrogenase A
NSCLC	Non-Small Cell Lung Cancer
ORR	Objective Response Rate
PDX	Patient-Derived Xenograft
PFS	Progression-Free Survival
ROS	Reactive Oxygen Species
scRNA-seq	Single-Cell RNA Sequencing
ST	Spatial Transcriptomic
TKIs	Tyrosine Kinase Inhibitors
TME	Tumor Microenvironment
Tregs	Regulatory T Cells
