# Supplementary material for: Atlas-Guided Nanocarrier Strategies Targeting Spatial NTRK2/MAPK Signaling in EGFR-TKI-Resistant Niches of Esophageal Squamous Cell Carcinoma
Source: Pharmaceutics. 2026 Jan 30;18(2):181. doi: 10.3390/pharmaceutics18020181 (PMC12944126; doi:10.3390/pharmaceutics18020181)
Supplement: Supplementary file 1 [file pharmaceutics-18-00181-s001.zip › pharmaceutics-3902839-supplementary.pdf]

Supplementary Table S1:

Dimension	Conventional models	SSI framework	Practical implication
Research assumption	Resistance is primarily driven by target mutations or single bypass pathways. *[11]*	Resistance emerges from **spatial heterogeneity** and **topological rewiring** of signaling within defined niches. *[41, 42]*	Shift from linear causality to **spatial network** mechanisms, aligning with observed intra-tumoral heterogeneity.
Analysis scale	Bulk or single-cell measurements interpreted without explicit spatial context. *[48]*	In situ analysis of signaling axes and ecological boundaries at tissue level (Visium/CosMx/Stereo-seq + imaging). *[41, 49]*	Makes **where** resistance occurs a first-class variable, improving explanatory power.
Data types	Bulk RNA-seq or scRNA-seq dominate. *[48]*	Multi-platform spatial multi-omics (Visium/CosMx/Stereo-seq) plus spatial proteomics, integrated with AI modeling. *[42, 49]*	Supports **niche detection** and atlas reconstruction across modalities.
Signaling logic	Linear downstream routes (EGFR→RAS/MAPK or PI3K/AKT) considered in isolation. *[11]*	Parallel/feedback coupling (e.g., TrkB→MAPK ↔ immune axes) emphasized and mapped in space. *[11, 41]*	Reveals **compensation + immune evasion** as co-localized drivers, guiding combos.
Target identification	Based on mutations/overexpression lists. *[11]*	Spatial co-enrichment and **synchronous activation** (e.g., $TrkB^{+}$/p-ERK^{+} regions) define actionable hubs. *[11, 42]*	Increases target **precision** by anchoring to niches.
Intervention strategy	Single-agent or empirically combined regimens. *[11]*	Atlas-guided dual-target/feedback nanodelivery tailored to niches (e.g., TrkB targeting + pERK-responsive release). *[42, 49]*	Moves from "drug-to-target" to **"drug-to-map"** delivery.
Validation systems	Predominantly cell lines or non-spatial mouse models. *[48]*	Literature increasingly employs **PDX/organoid** systems with **spatial endpoints** to probe mechanisms. *[41, 42]*	Enables visualization of **signal decay** and **immune remodeling** in situ.
Model application	Explanatory/post-hoc mechanism narratives. *[11]*	Predictive navigation via spatial AI (e.g., SpaGCN) for niche detection and risk stratification. *[45, 49]*	Supports **patient-specific** planning and trial design.

Limitations	Lacks spatial resolution and real-time feedback. *[48]*	Dependent on spatial data quality, cross-platform standardization, and interpretable AI. *[42, 49]*	Highlights infrastructure needs for clinical translation.

Supplementary Table S2:

**Cohort / Platform**	**Sample size (cases / slices)**	**Resolution / Probes**	**Median effective capture**	**Notes**
**ESCC-Visium (Discovery cohort)** [49]	18 / 24	55 μm spot	≈ 4,950 genes per spot; ≈ 3,200 spots per slice	Primary surgical resections analyzed with Visium; mixed FFPE and fresh-frozen samples; SCTransform + Harmony for QC and batch correction.
**ESCC-Visium (Validation cohort)** [41]	10/12	55 μm spot	≈ 4,720 genes per spot; ≈ 3,050 spots per slice	Independent multi-center validation dataset; unified preprocessing and Cosine normalization workflow.
**CosMx SMI (Targeted panel)** [49]	6/6	Sub-cellular	≈ 300 targets per round × multiple cycles	Targeted spatial transcriptomics including TrkB and p-ERK panels for signal-axis profiling.
**Stereo-seq (PDX model)** [42]	4/8	Sub-cellular	Platform-reported capture statistics	Spatial multi-omics profiling of EGFR-TKI resistant PDX tumor slices to map compensatory niches.
**Organoid-based synthetic atlas** [41]	8/8	Single-cell aligned	–	Organoid + immune-cell co-culture model with time-series imaging to reconstruct immune–signal interactions.

Supplementary Table S3:

**Module**	**Method / Parameter**	**Key settings & references**
Normalization & batch correction	SCTransform; Harmony; cross-check with limma	vst features = 3,000; within-slice cosine normalization; Harmony theta = 2, lambda = 1; limma used for differential checks [122-124].
Spatial graph construction	kNN + radius adjacency; auxiliary Giotto neighborhood graph	k = 8; r = 100 μm; Giotto neighborhood radius ≈ 120 μm [113].
Spatial module detection	Hotspot; Giotto spatial modules	Gaussian kernel σ ≈ 1.5 neighbors; FDR < 0.05 [112, 113].

Joint embedding for domain refinement	SpaGCN	3-layer GCN; hidden = 64; epochs = 500; lr = 1e-3; weight decay = 1e-4 [45].
Niche scoring (CSC)	CSC-score = mean z-score of *CD44, ALDH1A1, SOX2, NANOG, PROM1*	Threshold: ≥ 90th percentile plus connected region ≥ 25 spots/cells; computed on SCTransform-normalized data [122].
Niche scoring (MAPK)	GSVA on KEGG_MAPK + p-ERK targets (*FOS, JUN, DUSP6, ETV4*)	MAPK-score ≥ 90th percentile; KEGG pathway reference for gene set [146, 155].
Niche scoring (Immune-cold)	TIL-score = mean(*CD3D, CD8A, NKG7, GZMB*) & inhibitory axis (*CD274, CD276, IDO1*)	Define TIL-score ≤ 10th percentile and inhibitory axis ≥ 90th percentile; immune abundance scoring framework [146, 156].
Overlap criterion	Jaccard overlap between Hotspot modules & SpaGCN communities	Jaccard ≥ 0.4 to call MAPK islands [45, 112].
Spatial significance	Moran's I / Geary's C with 1,000 coordinate permutations	Retain domains with FDR < 0.05; implemented in Squidpy-style workflows [149].
Robustness checks	block-CV (500 μm blocks); LOPO (leave-one-patient-out)	Spatial blocking & subject-level CV for generalization [150, 157].
Resolution & registration stress tests	Down/upsampling (±25% coverage); sub-pixel jitter	Evaluate Dice/IoU overlap stability under perturbations [150].
Multimodal co-localization	Dual-IF TrkB / p-ERK co-localization with Pearson's r; Ripley's K for spatial point pattern	Threshold Pearson r ≥ 0.3; windowed (100 μm) agreement with transcript-level GSVA maps [158, 159].
Cross-modal alignment	CCA + MNN anchors	Seurat CCA/anchors and MNN for cross-batch/modal alignment [126, 160].
External biological validation	PDX / organoid literature patterns for TrkB$^+$/p-ERK$^+$ area, CSC markers, CD8$^+$ infiltration	Paired Wilcoxon and Cliff's delta to summarize effect sizes in reported preclinical settings [41, 49].

Supplementary Table S4:

**Endpoint**	**Model / Comparison**	**Outcome (median [IQR])**	**Statistical test & Significance (FDR<0.05)**
Tumor growth inhibition rate (TGI, %) [41]	PDX: TrkB/pERK dual responsive nano vs control	65–72 vs 0–10	Paired Wilcoxon test (FDR<0.05)

$TrkB^+$/$pERK^+$ area change (%) [11]	PDX: dual responsive nano	−45 ~ −58	Permutation test (FDR<0.05)
CSC marker reduction (ALDH1A1/CD44, %) [235]	PDX: dual responsive nano	−30 ~ −45	Cliff's delta = medium–large effect
$CD8^+$ T-cell infiltration increase (fold) [245]	PDX: dual responsive nano	1.6–2.3×	block-CV consistent effect
CD276 expression reduction (%) [231]	PDX: dual responsive nano	−25 ~ −40	FDR<0.05
IC_{50} reduction (%) [238]	Organoid: dual nano vs free drug	−40 ~ −55	Mann–Whitney test (FDR<0.05)
Penetration depth increase (%) [252]	Organoid: dual nano	+35 ~ +50	Dice/IoU ↑ (FDR<0.05)
Safety (ALT/AST, CBC) [231]	PDX: all groups	No significant difference	Multi-comparison non-significant

Supplementary Table S5:

**Barrier / Challenge**	**Clinical relevance**	**Potential mitigation strategy**
Cross-site variability in spatial data and batch effects [122, 123]	Niche calling and patient selection may shift across platforms/centers.	Unified QC/normalization (SCTransform), batch harmonization (Harmony), and reporting of platform metadata; multi-center concordance studies [49, 149].
AI model generalization and interpretability [150, 157]	Risk of overfitting and data-leakage undermines decision support.	Spatial block-CV / LOPO validation; model cards and transparent reporting; sensitivity analyses to staining/registration noise [49].
EPR variability and human PK/PD uncertainty [326]	Mouse–human gap reduces delivery to resistant niches.	Biomarker-anchored inclusion (e.g., TrkB/pERK atlas signatures), image-based pharmacology, and adaptive dosing; rigorous reporting per MIRIBEL [327].
Immunogenicity and off-target sequestration [326]	RES uptake and immune activation may limit efficacy/safety.	Surface engineering (stealth coatings), ligand density optimization, and stepwise FIH dose-escalation with immune monitoring [327].
CMC/GMP scale-up and batch consistency [327]	Reproducibility of ligand conjugation/linker stability is critical for approvals.	Early CMC alignment, stress/stability testing, release specs (size/PDI/zeta/ligand copy number), and reference materials [49].
Regulatory and trial-design complexity [328]	Companion diagnostics and complex endpoints	Phase 0/early window-of-opportunity trials with spatial endpoints; pre-specified success criteria for niche engagement and immune remodeling [49].

	complicate pathways.	
Data privacy and multi-institutional model training [280]	Sharing raw multi-omics/WSI may be restricted.	Federated/secure learning, differential privacy, and on-site model evaluation frameworks to enable external validation [149].

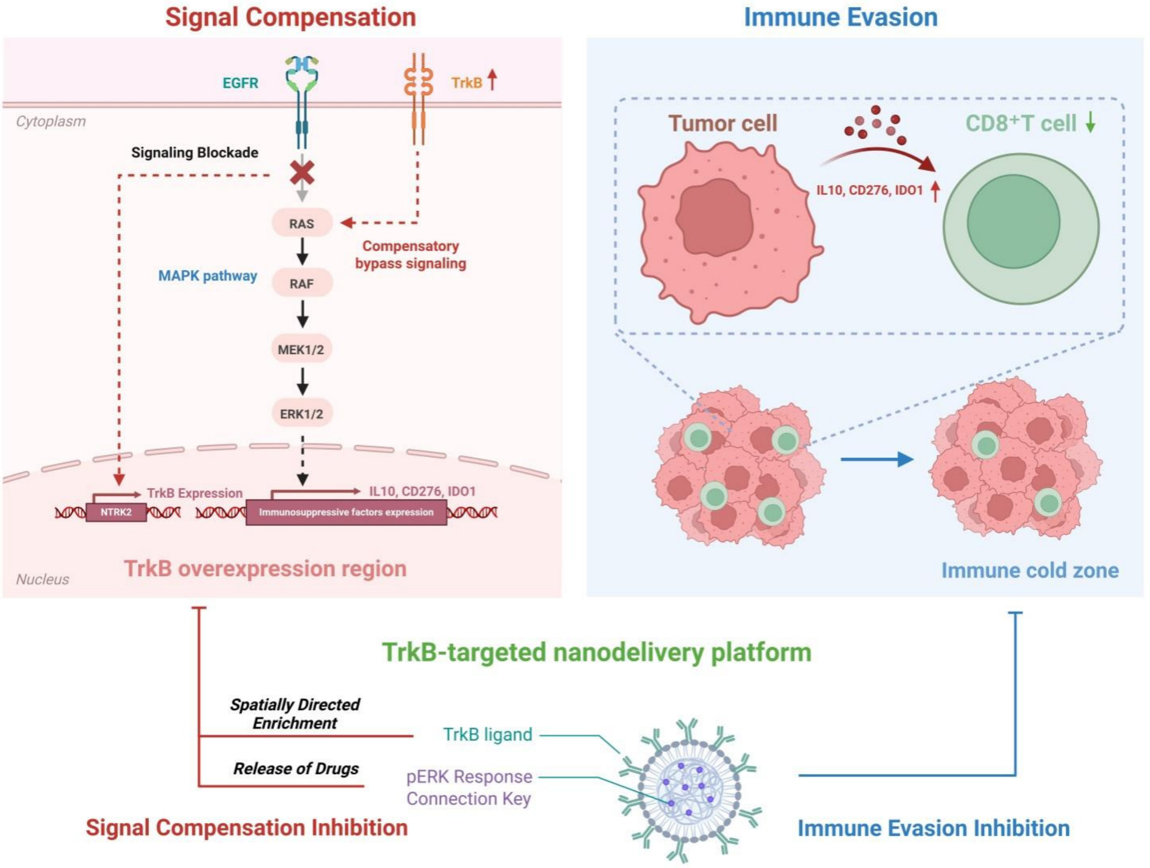

Supplementary Figure S1: *Created in BioRender. T, J. (2025) https://BioRender.com/dkr4iyw*
